# The relationship of the source of punishment and personality traits with investment and punishment in a public goods game

**DOI:** 10.1038/s41598-024-71106-x

**Published:** 2024-09-09

**Authors:** Johannes Rodrigues, Natasha Leipold, Johannes Hewig, Grit Hein

**Affiliations:** 1 Department of Psychology V: Differential Psychology, Personality Psychology and Psychological Diagnostics, Julius-Maximilians-University of Würzburg, Würzburg, Germany; 2 Department of Translational Social Neuroscience, University Hospital Würzburg, Universitätsklinikum Würzburg, Würzburg, Germany

**Keywords:** Psychology, Human behaviour

## Abstract

In this study, we investigated the motivations behind punishing individuals who exploit common resources, a phenomenon crucial for resource preservation. While some researchers suggest punishment stems from concern for the common good, others propose it is driven by anger toward free riders. To probe these motivations, we developed a modified public goods game in which participants had the option to use their own money or the money from the common pool to punish free riders. The analysis included choice behavior, mouse trajectories, and personality traits like anger, empathy, and altruism. According to our results, investments were highest, and punishment was strongest if participants could punish using credits from the common pool, indicating that this is the preferred option to diminish free riding and maintain cooperation in public goods and common good contexts. Also, punishment was highest if the punisher’s own investment was high, and the investment of others was low. Concerning traits, highly altruistic individuals tended to invest more and punish less in general but gave harsher punishments when they did choose to use the common pool punitively. Conversely, trait anger and trait empathy were linked to low investment while trait empathy also tended to be related to lower punishment. Taken together, these findings underscore the role of situational factors and personality traits in fostering cooperative behavior and shaping societal norms around costly punishment.

## Introduction

In all societies, humans rely on shared resources, or "public goods," that range from natural resources such as fresh air to institutional resources such as public transportation and schools. The maintenance of these public goods requires the participation of all members of a society. While many individuals contribute their own resources to maintain and enhance public goods, others exploit these resources without contributing anything themselves^1–3^. This observation is supported by solid empirical evidence obtained from common good or public goods games^4^. In the basic version of these games, participants are asked to contribute private funds (such as points or token money) to a common pool^1,3^. The amount contributed by all players is then multiplied by a factor greater than one but less than the number of players. The resulting sum is paid out as a "public good" to all participants, regardless of whether they contributed to the pool or not^4^. As a result, players can benefit from the public goods without contributing, and this free-riding or exploitation often leads to personal maximal gain^1^. This results in a dilemma: everybody collectively achieves the maximum gain if everybody fully contributes all their resources to the public goods, but each individual achieves the best payoff by contributing nothing to the public goods while benefitting from the other group members’ contributions^5^.

Studies reveal varying individual tendencies in contributing to public goods. Some are cooperators, contributing to the common good, while others are free riders, exploiting it without contributing. Conditional cooperators contribute if others do likewise and exploit if others do so too. However, as time progresses, the number of free riders tends to rise. Conditional cooperators eventually join the exploitation, resulting in a collapse of the cooperation essential for sustaining the public good^2,5^.

Introducing punishment to the public goods game has been shown to be an effective way of maintaining cooperation^5,6^. In this context, participants can invest their own credits or money to punish those who did not contribute to the public goods. The results of these studies show that introducing punishment increases the likelihood that free riders contribute to the public goods, while cooperators maintain their level of cooperation. Interestingly, despite their differences in social preferences (i.e., the preference to cooperate or defect), cooperators and former free riders invested a similar amount of resources to punish players who deviated from their own contributions^5,6^.

There is evidence that the extent to which participants punish free riders is related to individual differences in anger, on state and trait level^5,7–11^. Alternatively, it has been suggested that individuals punish to maintain cooperation^12,13^. In this case, empathy may play a role in addition to anger over the deviation from the expectation of cooperation, as empathy has been associated with the goal of increasing cooperation to benefit the welfare of others, such as those who are exploited by free riders in the public goods game^14–18^. Another overarching motivation very closely related to empathy that may drive the punishment behavior is altruism^19^. This motivation favors prosocial behavior that helps others without imposing additional harm, if possible^8,9,20^. In contrast to anger, which is clearly associated with punishment, altruism and empathy may only partly be linked to punishment if other options are available^8,9^ and in specific cases, even reduced punishment is to be expected.

Taken together, existing evidence suggests that punishment in public goods games may stem from either anger towards defectors or concern for the common good and other players, stemming from motivations like altruism and empathy. To distinguish between these driving forces, it is necessary to create a paradigm that gives participants one option to punish at the expense of the public goods (aimed at venting anger, disregarding others' welfare) and another option to punish while preserving the public goods (aimed at punishing defectors to motivate prosocial behavior). To date, no study has compared these simultaneous punishment options to elucidate the motive behind punishment in public goods games.

To rectify this, in the current study, we used two different types of punishment options: one in which participants could pay to punish defectors with their own money (own-money-punishment option^5^) and another in which participants could pay for punishment using money from the common pool (pool-money-punishment option). In the first option (own-money-punishment), they punished entirely at their own cost and spared the common pool resources. In the second option (pool-money-punishment), punishment reduced the overall size of the common pool, but the participants still received their respective shares. Hence the punishment in this option is "at society’s expense"—causing an overall decline in the public/common good for all other players, without cost to the punisher themselves. The reasons for including these experimental conditions were as follows: the own-money-punishment option was implemented to enable the participants to punish in the same manner as in previous public good games^5^, and to offer the participants a chance to assign punishment without hurting anyone but themselves and the defector. The pool-money-punishment option was implemented to allow punishment without using the punisher’s own resources. As punishment from the common pool would still reduce the size of the payout the punisher would get from the shared pool in the end, we opted to account for this by giving the punisher the same payout they would have gotten if no punishment had been taken from the common pool. This meant the contrast between the two punishment conditions was that the punishment was taken entirely from oneself or entirely from others. We also included a choose-punishment option, where participants could choose between using their own money or the common pool money to punish defectors, giving them the option to indicate which punishment method they preferred. These experimental conditions were also designed to disentangle the motivational aspects given by different personality traits. It was hypothesized that high anger would be linked to more punishment even at the expense of the public goods, while altruism and empathy would be linked to taking the preservation of the public goods into consideration.

Depending on the experimental condition, participants were presented only with the own-money-punishment option (own-money-punishment condition), only with the pool-money-punishment option (pool-money-punishment condition) or could choose between the two punishment options (punishment-choice condition). Additionally, we included a condition where no punishment was possible^5^. Besides the different punishment options, we manipulated the investment of the other players to simulate different types of commitment to the common good. The other players were fictitious, unbeknownst to the participants.

To gain further insight into the motivational processes that drive participants' punishment decisions, we measured their mouse trajectories. Mouse trajectories can reveal processes such as response conflicts, self-control, and certainty of response^21–25^. In contrast to classical behavioral variables (i.e., punishment and punishment source), this measurement is an implicit measurement that may provide additional information about the decision processes and their certainty^21–25^. Higher response conflict and uncertainty can be seen by either changing directions in the movement of the mouse or having a bias to one option while still moving to the other. In both cases, the geometric area between the direct path and the observed trajectory is enlarged. Moreover, we measured personality traits such as anger^26,27^, empathy^28–30^, and altruism^19,31–33^.

First, we predicted that any punishment option would increase investment (a summary of all hypotheses can be seen in Table 1). In addition, we hypothesized that the general punishment amount would be amplified if other players invested less or when one's own investment was higher.

**Table 1:** Hypotheses depending on the behavior of the participant and on the experimental design (causal) or personality traits (correlational).

Hypothesis type	Hypotheses	Participant behavior
Causal	More investment if punishment available	Investment
	From pool if less investment of other players	Punishment source
	From pool if more own investment	
	More punishment if less investment of other players	Punishment amount
	More punishment if more own investment	
	Certainty of decision is high if more own investment	Punishment source choice certainty
	Certainty of decision is high if less investment of other players	
Correlational	More investment for high trait benevolent altruism	Investment
	Less investment for high trait anger	
	More investment for high trait empathy	
	Less investment for high trait empathy	
	From oneself for high trait benevolent altruism	Punishment source
	From pool for high trait anger	
	Less punishment in case of high trait benevolent altruism	Punishment amount
	More punishment in case of high trait anger	
	Less punishment in case of high trait empathy	
	More punishment in case of high trait empathy	
	Certainty of decision high for high trait anger (pool)	Punishment source choice certainty
	Certainty of decision high for high trait benevolent altruism (oneself)	

Second, we hypothesized that participants would prefer the pool-money-punishment option if punishing free riders was mainly associated with anger (allowing punishment without primary concern for the public good). The effect of anger should be captured by the association of individual differences in trait anger scores on punishment choice^8,9^ and should be stronger if the other player invested relatively little in the common pool and the participant’s own investment was high. Accordingly, we predicted that the frequency and certainty (captured by mouse trajectories) of choosing the pool-money-punishment option would be positively related to trait anger scores and the participant’s own investment^34,35^, while it would be negatively related to the investment of the other players. Independently of the source of punishment, we predicted an increase in punishment amount with increasing scores in trait anger^8,9^. Also, we hypothesized that trait anger would be linked to less investment in the pool.

Third, we assumed that participants might prefer the own-money-punishment option if punishing free riders was mainly associated with the concern for others/the common good. Concern for others should be captured by individual differences in trait altruism and the effect should be even stronger if the free rider did not invest much in the common pool. Yet the influence of the participant’s own investment in the pool would only be expected to matter to the extent it was a limiting factor for the available punishment money. Accordingly, we predicted that the frequency and certainty of choosing the own-money-punishment option should be positively related to trait altruism scores and interact with the other players’ investment, being higher when their investment was lower. Independently of the source of punishment, we predicted a decrease in punishment amount with increasing scores in trait altruism^8,9^. Also, we hypothesized that trait altruism would be linked to higher investment in the pool.

Fourth, we expected trait empathy could be associated with either more or less investment, depending on the target of the empathy. If empathy is directed towards potential victims of a punishment, less investment and/or lower punishment may be expected, whereas if it is directed towards victims of an exploited public good, we might expect higher investment and/or higher punishment.

Finally, we predicted that the different punishment options and individual differences in personality traits may affect the certainty of the punishment decision, captured by mouse-trajectories and more specifically the deviation from the ideal line that could be taken to the option that is chosen. For example, the certainty of the punishment decision may be higher in the pool-punishment option compared to the own-punishment option and in participants scoring high on trait anger.

## Results

**Fig. 1:**
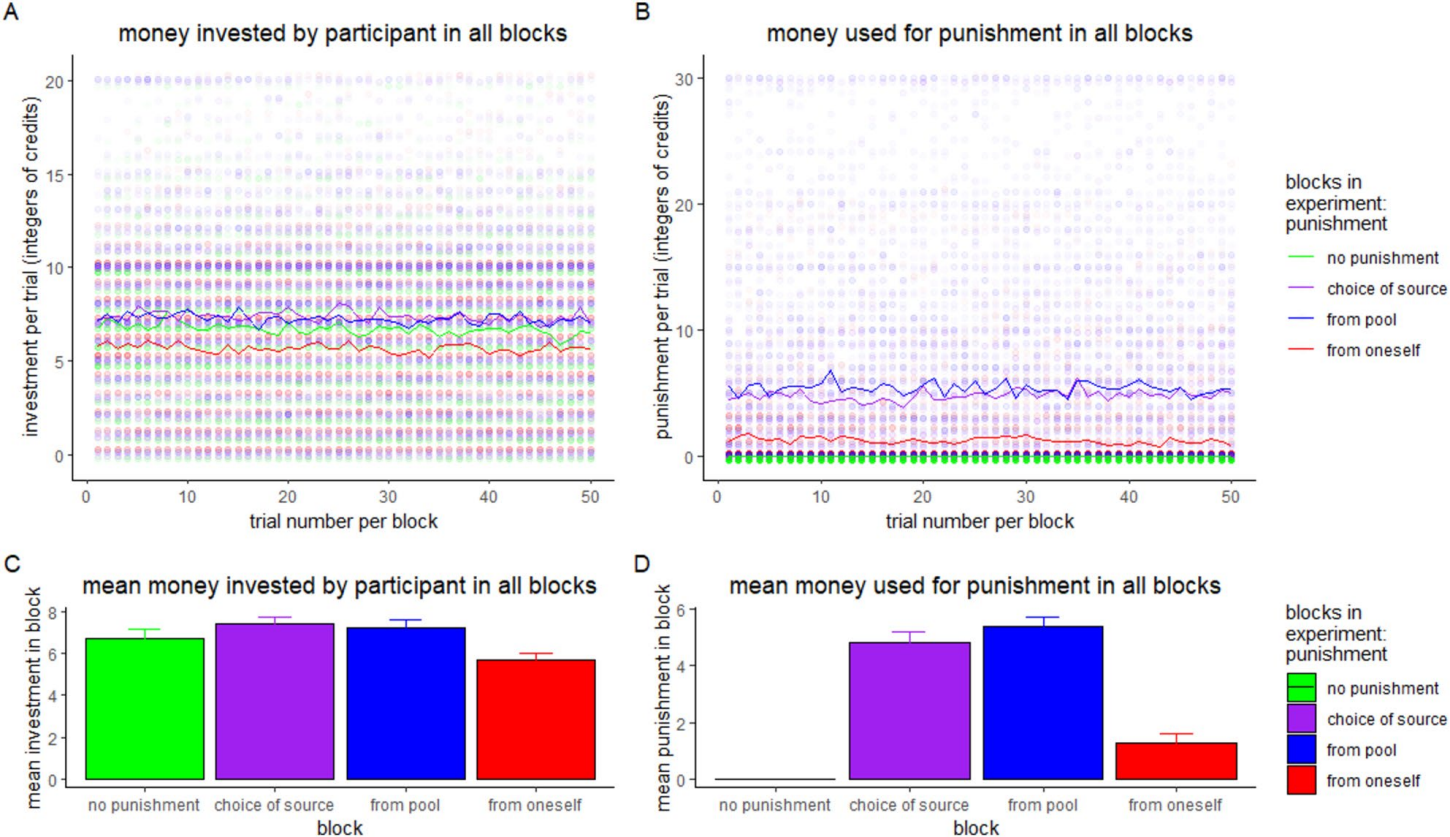
Money invested and used for punishment for each trial by the participants. Panel (**A**) displays the investment in each trial in each block (thickness of the circles indicates number of occurrences, colored lines display the mean investment per trial), panel (**B**) shows the money used for punishment in each trial in each block (thickness of the circles indicates number of occurrences, colored lines display the mean punishment per trial), panel (**C**) shows the mean money invested by the participants per block (error-bars show mean within SE), and panel (**D**) shows the mean money used for punishment per block (error-bars show mean within SE).

**Fig. 2:**
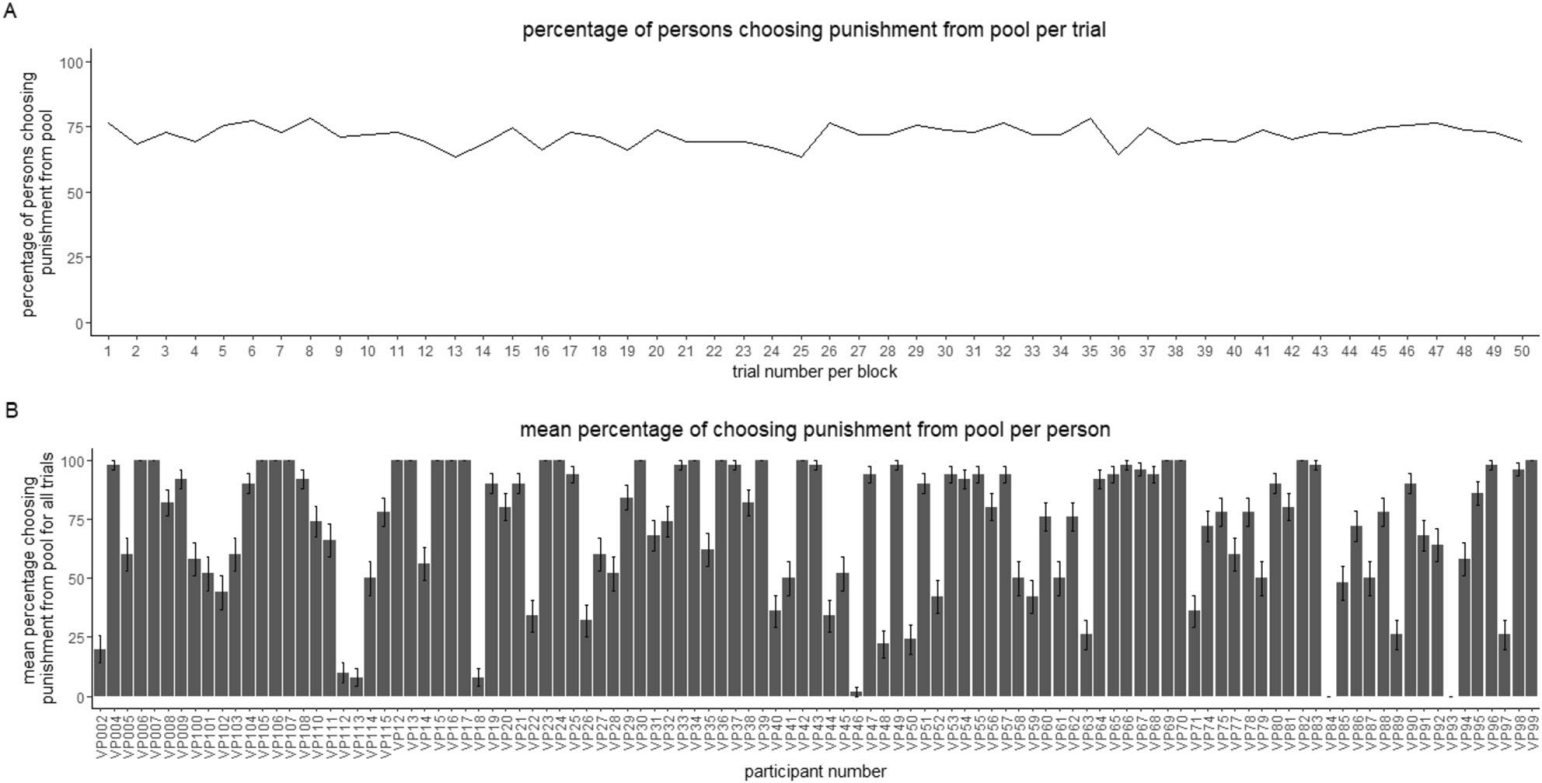
Percentage of choosing punishment from pool in the block where the participants were able to choose the punishment source. Panel (**A**) shows the percentage of persons choosing punishment from pool per trial, panel (**B**) shows the mean percentage of choosing punishment from pool per person (error-bars show SE).

The investment and the punishment given in all blocks over all trials can be seen in Fig. 1 and Table 2. For the amount of punishment and investment there was an effect of trial number (see table S4 and S5 for model selection and table S6b and table S6e in the supplemental materials for model details), with punishment and investment slightly declining over time. However, the decline is not at the same rate for all conditions but specific to the conditions (see Table 2). In Fig. 2, the percentage of persons choosing punishment from pool per trial (Fig. 2 panel A) and the mean percentage of choosing punishment from pool per person are shown (Fig. 2 panel B).

**Table 2:** Punishment and investment of participant depending on blocks: Correlation with trial and percentage of punishment choices.

Block	Correlation of participants’ investment with trial number	Correlation of investment of total amount of punishment with trial number	Percentage of punishment from pool (%)
No punishment	− 0.0376		
Punishment self	− 0.0148	− 0.0332	0
Punishment pool	− 0.0167	− 0.0066	100
Punishment choice	− 0.0190	0.0186	72

### Causal hypotheses

#### Investment in the pool

The possibility of punishing the other participants led to more investment compared to when no punishment option was given at all (mean investment = 6.693, SD = 4.219), if the punishment was given from the common pool (β = 1.778, *z* = 6.96, *p* < 0.001, mean = 7.24 SD = 4.47) or could be chosen (β = 1.566, *z* = 6.13, *p* < 0.001, mean = 7.381 SD = 4.584). If the punishment had to come from the participant’s own resources, no difference was found (β = 0.092, *z* = 0.359, *p* = 0.72, mean = 5.60 SD = 3.974). Thus, the expectation of higher investment if punishment was available could partly be supported. For further details of the influences on investment see supplemental table S6e.

#### Source of punishment

The amount of the participant’s investment in the present trial could predict the source of punishment successfully, with higher investment from the participant leading to punishment from the pool (β = 0.108, *z* = 6.681, *p* < 0.001) as predicted, while higher investment from the others led to punishment from the participant’s own resources (β = − 0.042, *z* = 8.501, *p* < 0.001). This also confirmed the second prediction about the choice of punishment increasingly coming from the common pool if others invested less money. For further details of the influences on the choice of punishment, see supplemental table S6c.

#### Amount of punishment

**Fig. 3:**
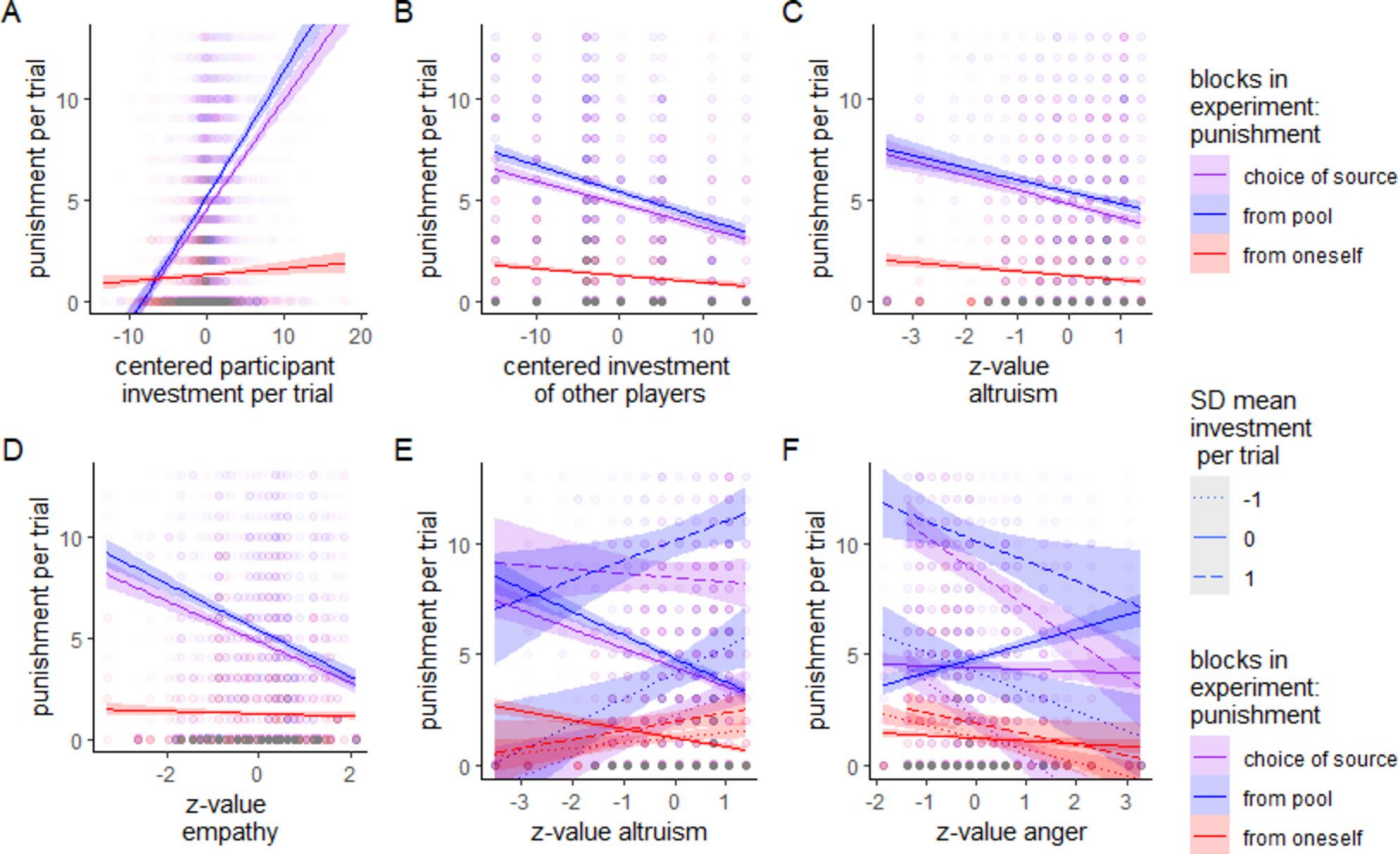
Amount of money invested in punishment depending (**A**) on the investment during the trial, (**B**) the centered investment of the players, (**C**) trait altruism, and (**D**) trait empathy, (**E**) trait altruism and mean investment per trial, (**F**) trait anger and mean investment of the participant per trial.

As expected, the general punishment amount was increased when other players invested less (β = − 0.097, *z* = 13.997, *p* < 0.001), and when one's own investment was higher (β = 0.438, *z* = 20.466, *p* < 0.001). Both effects were amplified in the choice block and in the pool block as shown in Fig. 3A and B (*p* < 0.05 for all comparisons; for a complete presentation of all results of the regression models see results in detail supplement S6b).

#### Certainty of punishment source choice

**Fig. 4:**
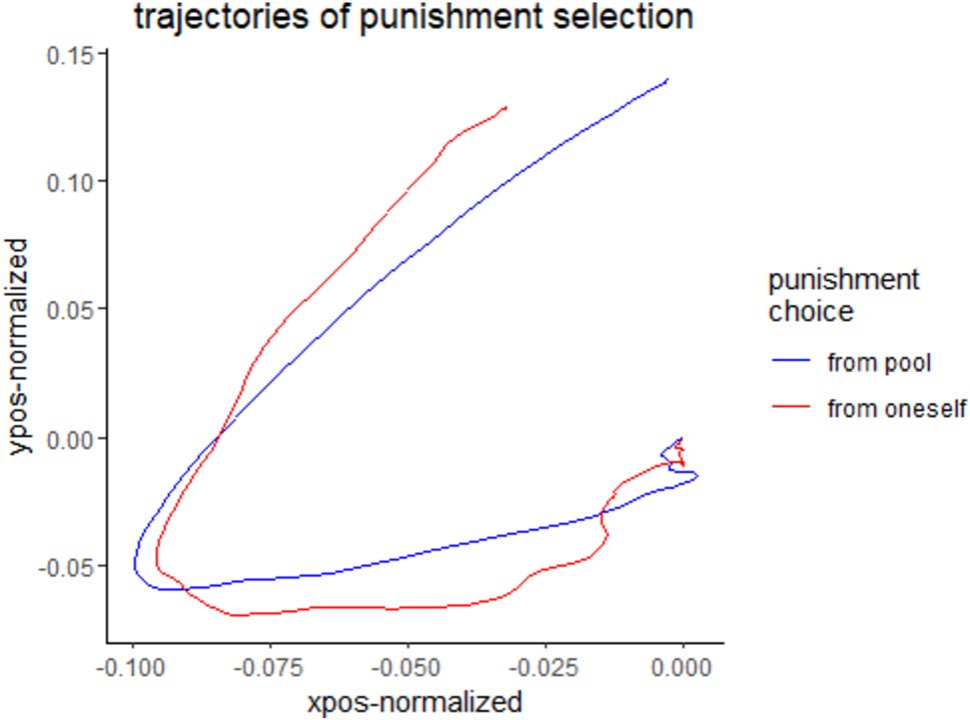
Trajectory of punishment selection. Depiction of the mean decision curve of the mouse to the relevant selection areas of the punishment source. In general, it was found that the punishment from pool led to a more certain decision curve (*p* < .05).

As predicted, certainty was generally reduced if others had invested more (β = − 0.001, *z* = 1.989, *p* < 0.05). In addition, although certainty of punishment was generally higher if one had invested more, as predicted (β = -0.002, *z* = 2.362, *p* < 0.05), this effect was reduced if punishment was taken from the pool (see Fig. 4; β = 0.002, *z* = 2.624, *p* < 0.01). The entire model can be seen in supplemental table S6d.

### Correlational hypotheses

#### Investment

**Fig. 5:**
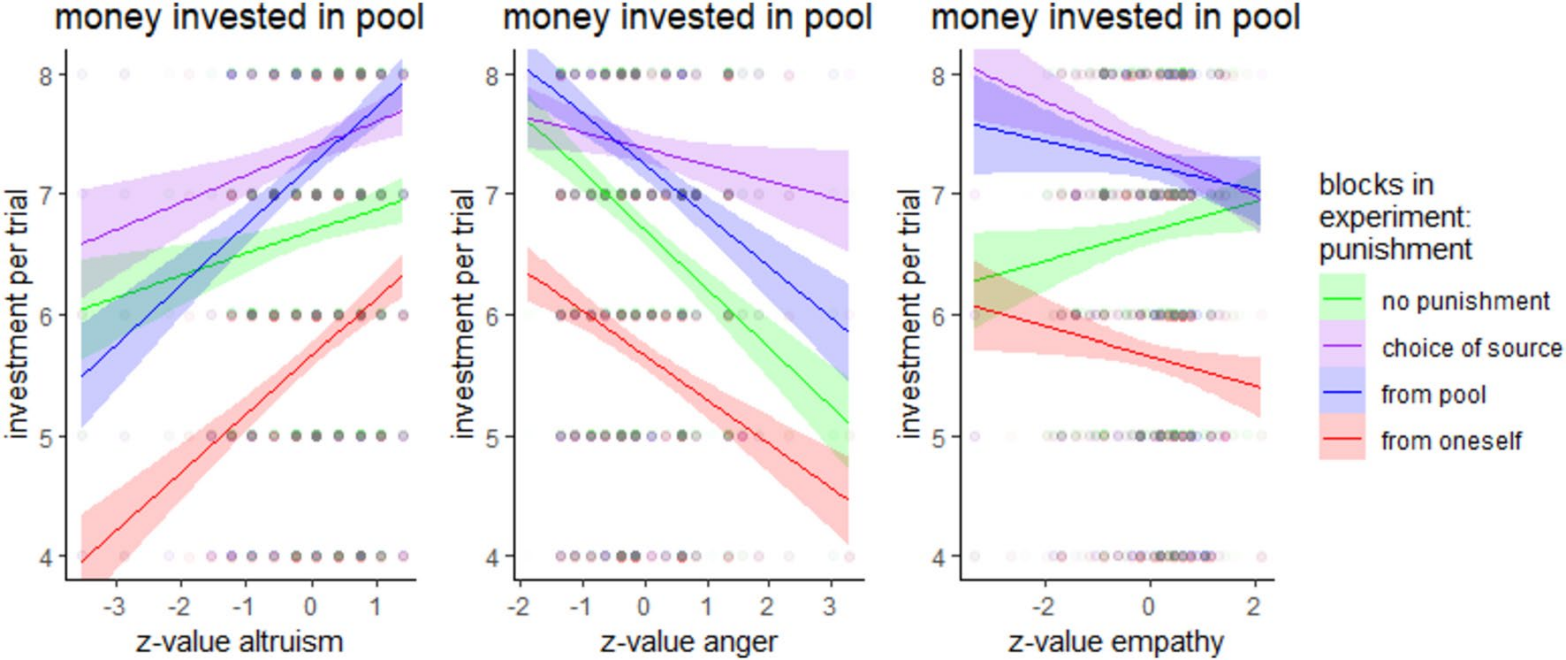
Mean investment in the pool dependent on trait altruism, trait anger and trait empathy for the different experimental blocks.

The influence of the traits could be seen for trait anger directly as a main effect (β = − 3.586, *z* = 3.267, *p* < 0.001), which reduced the investment as predicted, and for empathy, which also reduced total investment (β = − 0.147, *z* = 2.754, *p* < 0.01). For altruism, no main effect could be found; however, interaction patterns were partly in the directions hypothesized: benevolent altruism led to marginally more investment in all cases of available punishment (pool: β = 1.659, *z* = 2.855, *p* < 0.01, choice: β = 1.035, *z* = 1.79, *p* = 0.073, self: β = 0.951, *z* = 1.648, *p* = 0.099). Interestingly, there were also interactions for anger and empathy, dampening the main effects (see also Fig. 5). For further details of the influences on the choice of punishment, see supplemental table S6e.

#### Source of punishment

No main effect of the proposed effects concerning the source of punishment and trait relations was found. The influence of benevolent altruism was only present if the players had a high personal investment, with punishment coming from the player’s own resources if altruism was present (β = − 0.033, *z* = 3.231, *p* < 0.01), thus showing an interaction with the investment of the participants instead of the proposed main effect. For anger, no effect was found. For further details see table S6c in supplement.

#### Amount of punishment

The influence of the traits could not be detected as main effects but were only partly present for specific interactions. For altruism, if the investment of others was high (β = − 0.08, *z* = 5.042, *p* < 0.001), or the investment of oneself was high (β = − 0.166, *z* = 3.948, *p* < 0.001), which happened frequently (see above), there was less punishment, confirming our expectations. However, if the punishment came from the pool, there was more punishment (β = 1.344, *z* = 5.304, *p* < 0.001) for high altruism, contrary to our expectations. The investment effects on punishment were dampened in later trials (see supplemental table S6b). For trait anger, no hypotheses concerning main effect or interaction with higher punishment could be supported, but less punishment could be detected for the own-money-punishment condition (β = − 1.665, *z* = 4.856, *p* < 0.001, although dampened for high own investment: β = 0.26, *z* = 2.911, *p* < 0.01) than for the two other conditions (punishment from pool and choice of punishment) as well as for the own investment (β = − 0.181, *z* = 3.117, *p* < 0.01). For trait empathy, no supporting main effect could be found, but the interactions revealed that there was less punishment from the pool (β = − 0.220, *z* = 12.501, *p* < 0.001), from oneself (β = − 0.042, *z* = 2.235, *p* < 0.05), with higher investment from others (β = − 002, *z* = 2.082, *p* < 0.05), with higher investment from oneself (β = − 006, *z* = 2.073, *p* < 0.05) and during later trials (β = − 003, *z* = 5.177, *p* < 0.001). A graphical illustration of the trait effects can be found in Fig. 3 and the entire model can be seen in table S6b in the supplemental materials.

#### Certainty of punishment source choice

The analysis did not find any main effect of the proposed variables concerning the certainty of punishment source choice. The influence of traits was only present if the pool was chosen as source of punishment, with trait anger being linked to lower uncertainty if the punishment was taken from the pool and the others had invested a high amount of money (β = − 0.002, *z* = 2.733, *p* < 0.01). This effect was even more noticeable if the player’s own investment was also high (β = − 0.0005, *z* = 1.773, *p* = 0.076). Trait empathy led to lower certainty and higher AUC (β = 0.001, *z* = 3.204, *p* < 0.01) if the punishment source was from the pool. The entire model can be seen in supplemental table S6d.

## Summary of results

For a summary of the results see Table 3.

**Table 3:** Hypotheses depending on the behavior of the participant and on the experimental design (causal) or personality traits (correlational), including the results summary.

Hypothesis type	Participant behavior	Hypotheses	Support for hypotheses
Causal	Investment	More investment if punishment available	Supported for all but the punishment from oneself
	Punishment source	From pool if less investment of other players	Supported
		From pool if more own investment	Supported
	Punishment amount	More punishment if less investment of other players	Supported
		More punishment if more own investment	Supported
	Punishment source choice certainty	Certainty of decision is high if more own investment	Supported
		Certainty of decision is high if less investment of other players	Supported
Correlational	Investment	More investment for high trait benevolent altruism	Only marginal in interactions
		Less investment for high trait anger	Supported
		More investment for high trait empathy	Not supported
		Less investment for high trait empathy	Supported
	Punishment source	From oneself for high trait benevolent altruism	Only supported in interaction with high investment of others
		From pool for high trait anger	Not supported
	Punishment amount	Less punishment in case of high trait benevolent altruism	Supported for high investment of oneself or others, not supported for punishment from pool
		More punishment in case of high trait anger	Not supported
		Less punishment in case of high trait empathy	Supported for all but punishment choice condition
		More punishment in case of high trait empathy	Not supported
	Punishment source choice certainty	Certainty of decision high for high trait anger (pool)	Only supported in interaction with high investment of others and choosing the pool condition
		Certainty of decision high for high trait benevolent altruism (oneself)	Not supported

## Discussion

In this study, we aimed to distinguish between different proposed motivations driving costly punishment of free riders, including whether they demonstrated concern for the common good or neglected it. To achieve this, we designed a paradigm where participants could choose between punishing at the expense of the public goods (e.g., to vent anger) or punishing at their own expense (to preserve the public goods). In addition to assessing investment and punishment behaviors, we analyzed the choice between punishment from oneself and punishment from the public goods, as well as the certainty of this choice using mouse trajectory data and the area under the curve when comparing actual to ideal trajectories^21,22^, in order to achieve an implicit measurement for decision certainty.

First, as expected all punishment options increased the amount of investment in the common pool, which partly replicates previous research indicating that cooperation increases when punishment is available^5,6^. Yet, this was not the case for the block in which punishment had to be taken from one`s own money. We also found that higher investment by the participant led to more punishment, which may reflect a strategy to optimize the overall financial outcome for the participants protecting their investments. In addition, lower investment by the other (fictive) players also led to higher punishment. Previous research has shown that cooperation is rewarded and defection is punished^5^, particularly when one's own contribution to the common pool is high.

Second, as predicted higher investment from the participants further increased the punishment amount and its certainty in the pool-money-punishment block (and the punishment-choice block, see Fig. 1). Furthermore, lower investment from other players significantly increased punishment more strongly if the punishment was taken from the pool. Taken together, investments were highest and punishment was strongest in the conditions where punishment was available from the common pool, indicating that this option is preferable to diminish free riding and maintain cooperation in public goods and common good contexts.

For trait anger, we had predicted that the frequency and certainty of choosing the pool-money-punishment option would be positively related to trait anger scores, and we hypothesized that trait anger would lead to less investment in the pool. As expected, high anger individuals contributed significantly less to the common pool. This is in line with previous research suggesting that anger is closely linked to distrust in negotiation contexts^36^. Accordingly, high anger individuals saved their own resources instead of contributing them to the common good. Interestingly, although we had hypothesized that they would punish more in general, no main effect was found. However, they showed less uncertainty when choosing punishment from the common pool when others had invested relatively high amounts of money. These findings are in line with the approach motivation component of trait anger, which may contribute to a higher level of certainty in decision making, even when faced with mitigating circumstances such as cooperation from other players^37,38^. Taken together, this pattern of findings supports the hypothesis that participants high in trait anger take a relatively selfish approach towards our public goods game but does not support the previously hypothesized preference for more punishment.

Third, for high trait altruism, the predicted higher investment in the pool was found in all conditions where punishment was possible. This is partly consistent with the concept of altruism promoting prosocial behavior^8,9,39^, but our results did not show higher investment in the common resource when punishment wasn’t an option, indicating that control over others might be an important factor. Independently of the source of punishment, we predicted and found a decrease in punishment amount with increasing scores in trait altruism^8,9^ when there was high investment from others or oneself. Importantly and as predicted, within the punishment-choice block, high altruists were more likely to choose punishment from themselves instead of from the pool when others had invested more. This aligns with the benevolent, harm-avoiding concept of altruism put forth by Rodrigues et al^8,9,39^. Yet, this harm-avoiding preference was not fully confirmed as the amount of punishment was also higher in the pool-punishment condition. Thus, participants with high trait altruism tended to punish free riding more from their own money when possible, suggesting that they may tolerate self-damaging behavior or costs^12,39,40^, but they still punished more than participants low in trait altruism if their only option was to use the common pool. This increase in punishment is not entirely consistent with the concept of harm-avoiding benevolent altruism^8,9^, but it underscores the concern that high-altruism participants had for the victims of defection. It has been shown previously that when only one reaction pattern is possible and no option to compensate victims is given, altruism leads to punishment behavior^8^. This could be the case in our study, as no other option was given to support further cooperation (e.g. compensation), and the need to help the other investing players may have overridden the urge to be benevolent, leading to an increase in punishment even when it was only possible from the common pool. Taken together, these results demonstrate that participants scoring high in trait altruism show a highly cooperative, prosocial and altruistic pattern that includes higher investments, and lower punishments in some contexts. Yet, they are also willing to give higher punishments to free riders in contexts where there may be no other available option to change behavior and therefore uphold the social contract^6^.

Fourth, in contrast to the relationship with altruism, high empathy predicted lower investment in the pool. Highly empathetic participants may avoid investing in a pool that is being used for punishment, because they may feel empathy for anyone targeted for punishment. This interpretation would be in line with the finding that participants with higher levels of empathy also showed increased uncertainty when selecting the punishment source, specifically when choosing the common pool as the source. This suggests that empathy may prompt individuals to hesitate when deciding about an option that is associated with higher levels of punishment. As expected, empathy was linked to lower levels of punishment, but only when it wasn’t possible to choose the punishment source. Given both investment and punishment were lower for participants high in trait empathy, these results suggest that empathy may not always lead to prosocial behavior, but rather to a greater sensitivity to the behavior of others, as noted in previous research^41^. This may have led to anticipation of low cooperation from the other players, and this might also explain low investments. Hence, the prosocial aspect^16,42^ that we were expecting from trait empathy in general could only be seen in rather specific parts of the complex task.

## Limitations

One limitation of this study is the complexity of the public goods game, especially when using only one human participant and employing deception about the other players. While this allowed us to control the investments made by other players, it may have influenced the participants' tendency to cooperate, especially when punishment was possible^5^. In addition, as we only controlled one of the important predictors of punishment given by the other players (the investment of the fictive players) and did not adjust it based on the participants’ investment, our models could only implement statistical control instead of experimental control over the absolute negative deviation^6^. Yet, controlling for both variables would have made the experimental design overly complex, so we settled for only a statistical control by adding the participant’s own investment into the punishment analyses. Moreover, the fact that only the participant was given the opportunity to provide punishment may have created a power imbalance, resulting in altered response patterns and less investment by the participants than expected^43^. Additionally, the participants' anticipation of punishment from others may also have influenced their behavioral decisions^5^. Finally, we used deception, because investigating the effect of trait differences in behavior in three different conditions with real interactions would have required a large sample, which we were not able to collect based on the existing funds. The overall results (e.g. decline in punishment and investment over time) replicate the findings of studies with public goods games that used real anonymous interactions^6,44–46^, indicating the credibility of the cover story. We nevertheless acknowledge that interactions between real players would have been preferable.

Second, as expected, the source of punishment had an impact on the amount of punishment, with the highest amount when punishment came from the common pool, the second highest amount if a choice of the punishment source was given and the lowest amount when punishment came from oneself. This does not necessarily mean that punishment is not aimed at maintaining the public goods but could also mean that the possible amount of punishment is greater when taken from the pool. Hence the punishment may be delivered to a greater extent when participants choose to use the pool money over their own money, especially since the amount of the player’s own investment limits the punishment option when their own money is chosen as the punishment source.

It is important to note that the study was not preregistered, which limits the confirmatory nature of the findings and emphasizes the exploratory aspect of the study. Furthermore, the sample size was estimated based on previous similar studies rather than a pilot study, which may have influenced the accuracy of the results. Although the participant pool was diverse, the study could have benefitted from a comparison of extreme groups based on relevant traits to further enhance the effect patterns. Therefore, future studies could use the effect sizes reported in this study to recruit participants and utilize extreme groups to obtain more robust effects.

Interestingly, we only observed trait influences on the investment part of the task when punishment was available. Furthermore, as previously noted, only a few select traits influenced the choice of punishment source. These effects may be attributed to the situational trait activation capacity of the experimental conditions and tasks. The lack of punishment in the original investment condition may have weakened the influence of the traits due to insufficient situational intensity or capacity for motivational induction, resulting in reduced trait interaction^47^. However, the conditions where punishment was available appeared to have a sufficient trait activation property, relative to the no-punishment condition. As such, some hypotheses regarding the traits could not be confirmed in terms of main effects, but interaction patterns emerged in the respective punishment conditions, that were previously hypothesized as main effects.

## Conclusion

In summary, our study explored the effects of punishment source, investments, and personality traits in a public goods game. We found that punishment from the common pool resulted in increased punishment, while punishment from oneself led to decreased punishment. Additionally, the source of punishment influenced investment behavior significantly.

To summarize the effects of different personality traits on behavior, we found that trait anger was associated with lower investment into the pool and more decisive punishment choices when punishment originated from the common pool. Conversely, trait altruism was linked to increased investment, decreased punishment in general but increased punishment if the source of punishment was from the pool, providing partial support for a prosocial, harm-avoiding, benevolent component of altruism, but also a rule-reinforcing component. High trait empathy participants exhibited lower investment in all conditions. They exhibited particularly low certainty of punishment and empathy was associated with less punishment. These findings emphasize the role of both situational factors and individual traits in fostering cooperative behavior and establishing costly punishment as a societal norm.

## Method

### Ethical statement

The study was carried out in accordance with the recommendations of “Ethical guidelines, The Association of German Professional Psychologists” with written informed consent from all subjects. All subjects gave written informed consent in accordance with the Declaration of Helsinki before they participated in the experiment. The protocol was approved by the local ethics committee of the department of psychology of the Julius-Maximilians-University of Würzburg (GZEK 2018-11).

### Participants

An a-priori sampling size calculation for the analyses was performed using an effect of *r* = 0.3 as the desired effect size. The calculation with G-power with alpha = 0.05 and power(1-beta) = 0.8 leads to a requirement of at least 82 participants per sample. In the final sample, 107 participants were recruited. Using Optimal Design Plus Empirical Evidence^48^ for an estimation of the detectable effect in the multilevel analysis with n = 107, α = 0.05, power (1 − β) = 0.8 and ρ = 0.1, we find an effect of δ = 0.17.

Participants were recruited through local advertisements. Participants were given course credits or a monetary compensation of 10€ independent from their actual monetary outcome. The compensation was given as if they would have played with the “optimal” strategy that would have preserved the maximum amount of money for them, to account for possible imbalances of payment due to participant behavior. This compensation method was only revealed to the participants after the paradigm was complete. While playing the paradigm, participants were told that their monetary investment and outcomes in the public good game would be their participation compensation. All participants were at least 18 years old, right – handed, non-color blind and with no history of any psychiatric disorder. The mean age was 27.57 (*SD* = 9.33) and 85 were female (79.44%).

### Paradigm

The study used a variant of the public goods game originally developed by Weber et al.^5^. In our version, participants were the only real players, while all other players were simulated by the computer. This is a deviation from the original work of Weber et al.^5^, as in their work, they used real players only for all positions. The setting of the experiment was a repeated one-shot game with fictitious opponents, where during each trial, the participant played with three (fictitious) opponents that were controlled by the computer, unbeknownst to the participant. While running real interactive experiments is preferable, in this study, we were interested in the effect of trait differences in behavior in three different conditions. To compare these effects, it was necessary to keep the behavior of the other players constant across conditions. Keeping the other’s players’ behavior constant across conditions in real life interactions would have required a huge sample size, which we were not able to test based on the existing funds. To circumvent this problem, we used a variant of the public goods game developed by Weber and colleagues^5^ where each participant was the only real player and all other players were simulated by the computer based on computer-generated investments. Every subsequent trial, the participant played with three different “opponents”. Hence, the participants could not know whether they had already played against any of the players during each trial and even if they ended up “replaying” with any specific player, it would not be clear to them that they did. This setup was used to ensure a controlled repeated one-shot game situation with absolute control over the different investment that was given by the “other players”, to investigate the behavioral responses to the investment as well as the relevant trait influences on the chosen behavioral responses. In addition, this setup minimizes the expectation of having a direct benefit from punishment in the next trials, compared to repeated game settings. Hence, the investment in punishment may be less influenced by strategic considerations and more likely to be driven by other motivational factors and traits. The deception was revealed to participants at the end of the experiment. To ensure that participants understood the task, they had to correctly indicate the appropriate amount of money for four players in three examples before the main experiment began. If they passed this test, they proceeded to the four different blocks of the experiment.

In the first block, the public goods game was played without any punishment option. The other blocks included one of three punishment options, which were counterbalanced across participants. The punishment options differed in the source of the punishment. In the first punishment condition, the money required for punishment was taken from the common pool, i.e., the pool in which every player had invested (pool-money-punishment option). In the second punishment condition, the money required for punishment was taken from the participant's own money available for the trial (own-money-punishment option). In the third punishment condition, participants could choose between punishing with the money from the common pool or punishing with their own money (choose-punishment option). Five practice trials were given at the beginning of each block to familiarize participants with the task.

**Fig. 6:**
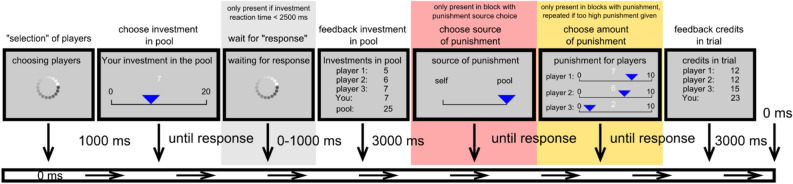
Schematic display of the different trial-types of the common good paradigm.

In each trial, participants experienced a waiting period of 1 s, representing the selection of the other players for that round (Fig. 6). They were then given 20 credits and asked to indicate via mouse click on a scale from 0 to 20 credits only containing integer credit options, how much money they wished to invest in that trial from their current credits in a common pool. The common pool was the sum of all players' investments, which was then multiplied by a factor of 0.4 and redistributed to all participants (lacking a division between the participants, but everyone got the amount of money), so their final amount of credits for that trial was equal to the redistributed sum plus the money they chose not to invest. No time limits were imposed on the investment decision. If participants responded faster than 2.5 s, they ostensibly waited for the response of the other players. Subsequently, participants were shown the common pool investment from each player (for 3 s). After this feedback, the final distribution of credits for each player in that trial was presented for 3 s.

In blocks where participants could choose the source of punishment (own money or common pool money), they decided between the two options via mouse click on the “self” or the “pool” option. Next, participants could punish the other players (except themselves) using a scale from 0 to 10 credits only containing integer options. If the participant spent more money than was available from the punishment source, they were given the respective feedback and had to redistribute the punishment, so it was less than or equal to the available credits. At the end of each trial, they received feedback about the amount of credits each player had, including themselves.

A schematic display of the different trial-types can be seen in Fig. 6.

Each block consisted of 50 trials with ten different fairness combinations concerning the three fictive players. The fairness combinations and the respective investment by the fictive players are shown in Table 4.

**Table 4:** Fairness combination of the fictive player.

Offer player 1	Offer player 2	Offer player 3	Cooperation category player 1	Cooperation category player 2	Cooperation category player 3
7	8	9	Middle investment	Middle investment	Middle investment
7	8	13	Middle investment	Middle investment	High investment
8	9	3	Middle investment	Middle investment	Low investment
7	2	12	Middle investment	Low investment	High investment
9	3	2	Middle investment	Low investment	Low investment
8	14	13	Middle investment	High investment	High investment
4	3	13	Low investment	Low investment	High investment
4	3	2	Low investment	Low investment	Low investment
13	12	4	High investment	High investment	Low investment
12	13	14	high investment	high investment	high investment

### Calculation of the credits

In the block without punishment, each player's earnings were determined by the credits they kept for themselves and the credits in the pool, which were multiplied by a factor of 0.4. If rounding was required for the pool, it was always rounded down, and participants were informed of this.

If participants were able to choose punishment from their own credits, they could use their own earnings from the current trial to punish other players. This reduced both the punished player's credits and the punishing participant's credits accordingly.

If participants were able to choose the punishment from the pool, the credits for the punishment were taken from the pool, and the calculation of the participant's credits was done as if no punishment was taken from there. This was to ensure that there were no costs for the punishment to the participant in this case.

Every participant was instructed that their real-world payoff would be equal to the total number of credits they had at the end of the experiment, multiplied by 0.75 cents. As mentioned above, this payoff metric was not actually used, but this was only revealed to the participants after participation.

Examples and illustrations of these credit computations are given in supplemental materials S1.

### Procedure

Before arriving at the laboratory, participants completed a web-based questionnaire using the online questionnaire platform SoSci Survey^49^ to assess relevant traits (see the trait measurement section) and demographic data such as gender, age, and handedness. Participants were then invited to the lab, with up to three participants being assessed during one session in one room, separated by a dash panel.

In the lab, participants were first asked to give (semi) informed consent after the experiment was described without revealing that the other players encountered in the paradigm were only fictive. They were then seated in front of a 61 cm (24″) widescreen monitor at a distance of 50–60 cm. A state measurement was taken, followed by instructions for the public goods game, and a confirmation that they understood the task by indicating the correct amount of money for each player for three examples. Subsequently, they performed the public goods game, with a different order of blocks for each participant, but with the block without punishment always being the first block. Following each block, state questionnaires were given, that are described in supplemental materials S1. After the experiment, the participants were debriefed. The paradigm was programmed and presented using PsychoPy2 (v1.90. ^3,50^).

### Apparatus

#### Trait measurement

For the measurement of trait altruism and prosocial tendencies, the Prosocial Tendencies Measure^31–33^ was used. To assess anger on a trait level, the German version of the State- Trait–Anger–Expression–Inventory (STAXI,^26,27^) was administered. The Saarbrücker Persönlichkeitsfragebogen (SPF; ^30^), a German version of the Interpersonal Reactivity Index^28,29^ was used for measuring empathy. For further traits that were measured for control purposes, i.e. trait anxiety, trait greed and big five personality traits, see supplemental materials S1, S6, method section.

### Statistics

All models were computed using R^51^ and the package glmmTMB^52^. The mouse trajectory was computed using the mousetrap package^22^.

#### Source of punishment

The choice of the source of punishment was analyzed with a hierarchical single trial multi-level binomial regression model in the block where the choice was possible. Higher values indicate punishment from the pool rather than from oneself on the dependent variable. The model included the trials on level 1 with the random slope trials for each person and the fixed effects of investment per trial by the participants and the other players. On level 2 the fixed effects of the mean investment of the participants and the traits altruism, anger, empathy, anxiety and greed were used. The trait variables on level 2 were grand-mean centered, and the variables on level 1 were person centered. The best model was chosen using the AICc and the probability of information loss (see Table S2 in supplement).

The mouse trajectories of the punishment source selection were analyzed with a hierarchical single trial multi-level regression model. As the dependent variable, the area under the curve was used as an implicit measurement of uncertainty of the choice^22^. The model included a random intercept for each person and the fixed effects of investment per trial by the participants and the other players as well as the punishment source that was selected. On level 2 the fixed effects of the mean investment of the participants and the traits altruism, anger, empathy, anxiety and greed were used. The trait variables on level 2 were grand-mean centered, and the variables on level 1 were person centered. The best model was chosen using the AICc and the probability of information loss (see Table S3 in supplement).

#### Amount of punishment

The amount of punishment was analyzed with a hierarchical single trial multi-level regression model. The model included the trials on level 1 with the random slope trials for each person and the fixed effects blocks of experiment (punishment choice/punishment pool/punishment self), investment per trial and the participants on level 2 with the fixed effects of the mean investment of the participants and the traits altruism, anger, empathy, anxiety and greed. The predictors on level 1 were centered within the participants, while the variables on level 2 were grand-mean centered. The reference category for the block effect was the punishment choice block. The best model was chosen using the AICc and the probability of information loss (see Table S4 in supplement).

#### Investment in the pool

The investment in the pool was analyzed with a hierarchical single trial multi-level regression model. The model included the trials on level 1 with the random slope trials for each person and the fixed effects blocks of experiment (no punishment/punishment choice/punishment pool/punishment self) and the participants on level 2 with the fixed effects of the traits altruism, anger, empathy, anxiety and greed. The trait variables on level 2 were grand-mean centered. The reference category for the block effect was the no punishment block. The best model was chosen using the AICc and the probability of information loss (see Table S5 in supplement).

#### Exploratory analyses

For exploratory analyses see S1 in supplemental materials.

## Supplementary Information

Supplementary Information 1.

Supplementary Table S2.

Supplementary Table S3.

Supplementary Table S4.

Supplementary Table S5.

Supplementary Table S6.

## Data Availability

The data is available on OSF: https://osf.io/cy76v/?view_only=abd79265b0c5491f9b3b3acfa86ea95b.
