# Supplementary material for: The relationship of the source of punishment and personality traits with investment and punishment in a public goods game
Source: Sci Rep. 2024 Sep 9;14:21046. doi: 10.1038/s41598-024-71106-x (PMC11385193; doi:10.1038/s41598-024-71106-x)
Supplement: Supplementary file 1 — Supplementary Information 1. [file 41598_2024_71106_MOESM1_ESM.docx]

# Supplemental materials S1: Trait anxiety and trait greed

# Introduction concerning trait anxiety and trait greed

Anxiety shares being a negative emotion with anger, however it is not associated with approach behavior, but with avoidance or conflict ^1^. Trait anxiety is hereby defined as the disposition to react with state anxiety in uncertain or ambiguous situations. Anxiety has been linked to uncertain and conflicting situations as a mechanism to rise arousal and an orienting response, accompanied by behavioral inhibition ^1^ and passive avoidance ^2^. High levels of trait anxiety have been shown to have a strong influence on the social life by using avoidance as coping strategy in social behavior ^3^. Accordingly, anxiety undermines the willingness to communicate, which leads to avoidance of social interactions ^4^. However, it has been shown, that in other economic decision paradigms like the ultimatum game, high-trait anxious participants with low self-esteem accepted unfair treatment more often ^5,6^. Also, the expectations of getting a rewarding and successful interaction is lower in highly anxious individuals ^7^. Following these findings, we expected that anxiety would lead to less investment as well as less punishment in general, as the expectation to get a rewarding interaction is lower, but the acceptance of unfair behavior may also be higher as the punishment may be seen as a social interaction that is normally avoided. For the punishment from pool, we expected especially low punishment, as the social interaction is affecting even more other players. Contrary to the rather risk avoiding influence we expected by anxiety, we were also interested in the influence of greed on the interaction behavior, as this personality trait has massive influence on human economic interactions (see e.g., ^8–14^.

Greed depicts egoistic behavior in the economic decision context and is defined in our context “as desire to get more at all costs, including the excessive striving for desired goods and the willingness to accept that such striving may be at the expense of others.” ^10^. It was shown that greedy people maximize their profit and keep more money for themselves ^8,9,12,15^. However, they also tend to take higher risks to get higher gains ^10^. Concerning cooperation behavior and unfair offers in cooperation, they tend to dismiss unfair offers more often ^11^. Integrating all these findings, we expected greed to lead to lower investments and exploitation of the other players in the desire to get more at all costs. Additionally, we expected punishment being very low if the source of punishment was oneself, as the punishment in this case would lead to costs that are not immediately repaid, being a repeated one-shot paradigm.

In addition to the effects proposed concerning trait benevolent altruism, trait anger and trait empathy, we predicted that individual differences in anxiety and greed may play a role in investment as well as partly in punishment behavior, because of the influence that has been shown by trait anxiety and trait greed on social interaction and interactions in other bargaining games like the ultimatum game ^3–10,12,16^. The results and discussion about trait greed and trait anxiety are only provided as supplemental materials, to streamline the present manuscript.

# Method

## Paradigm

### Calculation of the credits

In the block without punishment, each player's earnings were determined by the credits they kept for themselves and the credits in the pool, which were multiplied by a factor of .4. For example, if Player 1 offered 5 credits to the pool, Player 2 offered 6 credits, Player 3 offered 7 credits, and the participant offered 7 credits, the pool would be 25. The resulting credits for each player would be calculated as follows: Player 1: 20-5+(25*.4) = 25, Player 2: 20-6+(25*.4) = 24, Player 3: 20-7+(25*.4) = 23, and the participant: 20-7+(25*.4) = 23. If rounding was required for the pool, it was always rounded down, and participants were informed of this.

If participants were able to choose punishment from their own credits, they could use their own earnings from the current trial to punish other players. This reduced both the punished player's credits and the punishing participant's credits accordingly. To illustrate this with the previous example and a possible punishment, suppose the punishment for Player 1 was 7, for Player 2 was 6, and for Player 3 was 2. The resulting credits distribution would be: Player 1: 20-5+(25*.4) -7 = 18, Player 2: 20-6+(25*.4) -6 = 18, Player 3: 20-7+(25*.4) -2 = 21, and the participant: 20-7+(25*.4)-7-6-2 = 8.

If participants were able to choose the punishment from the pool, the credits for the punishment were taken from the pool, and the calculation of the participant's credits was done as if no punishment was taken from there. This was to ensure that there were no costs for the punishment on the participant in this case. Using the same example as above, if the punishment source was the pool and the punishments were the same, the resulting credits distribution would be: Player 1: 20-5+(10*.4) -7 = 12, Player 2: 20-6+(10*.4) -6 = 12, Player 3: 20-7+(10*.4) -2 = 15, and the participant: 20-7+(25*.4) = 23.

## Apparatus

### State measurement

During the experiment before and after the different blocks, the Positive and Negative affect schedule ^17,18^ as well as the SAM rating scales ^19^ were used. However, they are not considered in the present analysis.

### Trait measurement

To assess trait anxiety, the German version of the State – Trait anxiety inventory ^STAI; 20^ was used. For the assessment of greed, the GR€€D scale ^12^ was used. For further explorative analyses, also the big five aspect scales ^BFAS, 21^ were assessed.

## Statistics

### Exploratory analyses

The trait interrelations as well as the mean investment and punishment for each block were investigated exploratory. Also, a t-test with Welch correction was performed to compare the amount of punishment in the different choices of punishment during the block where punishment was available.

The *p*-values were not adjusted in these analyses, due to the exploratory character.

# Results concerning trait anxiety and trait greed

## Invested money in the pool: influence of trait greed and trait anxiety

Anxiety marginally led to higher investment in general (β = 1.612, *z* = 1.869, *p* = .062). Greed did not lead to any significant investment difference (although on descriptive level, there was a tendency to invest less in general: β = -.501, *z* = 1.183, *p* = .237). A graphical illustration of the effects can be seen in Figure 1S below. The entire model can be seen in table S6 in supplement.


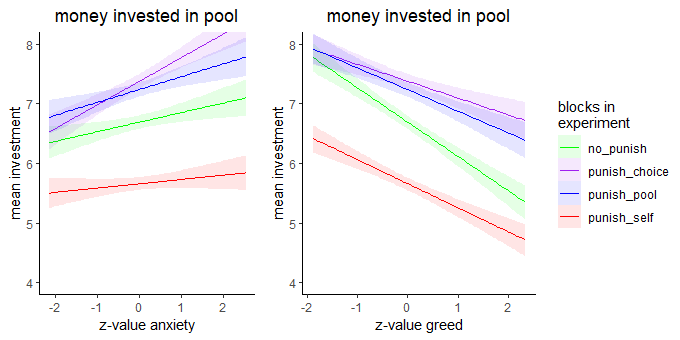


Figure 1S: Mean investment in the pool dependent on anxiety and greed for the different experimental blocks.

## Source of punishment

### Mouse trajectory: influence of trait anxiety

High trait anxiety led to a lower AUC and higher certainty in this case (β = -.013, *z* = 2.288, *p* < .05), but to slightly higher uncertainty, if the investment of the others and oneself was high (β = .0004, *z* = 2.12, *p* < .05). The entire model can be seen in table S8 in supplement.

## Amount of punishment: influence of trait anxiety and trait greed

The influence of anxiety on the amount of punishment was shown in interactions with the participants´ investment per trial, leading to an amplification of the investment effect leading to more punishment (β = .142, *z* = 2.838, *p* < .01, although being dampened for the pool punishment condition (β = -.272, *z* = 3.694, *p* < .001) and the punishment self-condition (β = -.156, *z* = 1.996, *p* < .05). In later trials, anxiety also led to more punishment (β = .036, *z* = 3.362, *p* < .01, although being dampened for the pool punishment condition (β = -.046, *z* = 6.286, *p* < .001) and the punishment self-condition (β = -.046, *z* = 6.199, *p* < .001, see Figure 2S panel A and for the own investment β = -.003, *z* = 2.958, *p* < .01)Also, anxiety interacted with the source of the punishment, dampening the influence of the punishment being from oneself (β = 1.72, *z* = 6.284, *p* < .001, see Figure 2S panel A).

Greed led to an amplification of the influence of the source of punishment being from oneself (β = -.306, *z* = 2.355, *p* < .05, see Figure 2S panel B). Additionally, higher greed led to less punishment, if the investment of the other players was high (β = -.019, *z* = 2.379, *p* < .05). This effect was inversed or dampened, if the punishment was taken from oneself (β = .038, *z* = 3.187, *p* < .01). Also, the amount of punishment per investment of the participant if the source of punishment was the common pool was amplified for high greed (β = .129, *z* = 3.591, *p* < .001).

The entire model can be seen in table S6b in supplement.


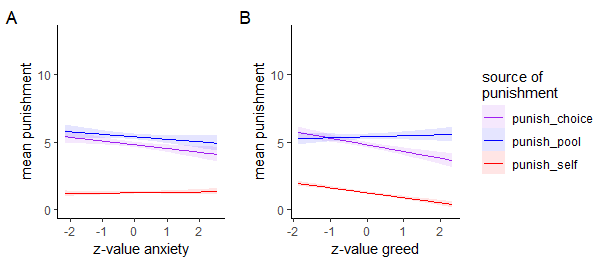


Figure 2S: Amount of money invested in punishment depending on the investment during the trial, the source of the punishment and the traits anxiety and greed.

# Discussion

## Influences of personality traits: trait anxiety and trait greed

For the interrelation of anxiety with the different parts of the task, we found for the investment a significantly different effect that the one we proposed. For high trait anxiety, we found higher investments in the pool. This effect may be due to the fear of social evaluation ^22^ or the avoidance of the risk of getting negative social evaluation ^23^. Hence, this risk aversion and fear of negative social evaluation may in this case lead to higher investment in the public good, instead of smaller interaction that was proposed by us due to the findings that avoidance of social interaction is prevalent in high anxious persons and that they expect lower rewards and success from their social interactions as other persons ^3,4,7^. Another interesting interaction of anxiety was given with the certainty of the punishment source choice. Here anxiety led to a higher certainty for the choice, even more so if the investment was high. One possible explanation would be, that person with high anxiety may have used a different strategy than other players, choosing thresholds and clear self-defined rules that enabled them not to ponder over the decisions but to execute them effectively. Yet, this explanation of this unexpected effect is only speculative. Concerning the amount of punishment that was given, anxiety led to lower punishment as expected, but this effect was not present for high participants´ investment in the present trial, if the punishment was taken from oneself or the choice of sources was given. Another deviation from the general tendency to dampen the punishment was given if the participants´ investment per trial was low and the source of the punishment was the pool. Then, the punishment was unexpectedly high. Generally, if the punishment was from oneself, the anxiety led also to a relative amplification of punishment. Despite these deviations, anxiety led to relatively lower punishment as expected. This pattern of findings may be due to the previously explained general fear of getting into social context ^4^ and also the higher acceptance of unfairness ^5,6^ and their lower expectation to get rewarding social interaction ^7^. These factors may all lead to lower punishment. However, if there is a choice of the punishment source or the punishment is to be given from oneself, high anxious persons might feel more confident to provide punishment, as they themselves also take a negative consequence and negative social evaluations by others might therefore occur less in their opinion.

The influence of greed on the investment of the participants could not be seen as proposed in a significant extend. This may be due to the experiment setting and task, being performed in a rather non-consume oriented environment or with a rather complex task. This may have hindered the influence of greed in this context ^10,24^, leading to less investment in the pool. However as expected, greed led to lower punishment, especially if the punishment was from oneself. In combination with the lower punishment if the other players also invest more, that was actually dampened if the own investment was higher, it fits very well the idea of greed as the tendency to get more ^12^ while valuing the input of other players. The notion of getting more at all costs ^10,12^ may be expressed by the small rise in punishment, if the own investment is rather high during one trial, but generally, high greed participants seem to “comfortably lean back and free ride”, although a lack in investment is not seen to a significant extend.

## Conclusion concerning trait anxiety and trait greed

Anxiety led to higher investments in the pool and higher punishment from oneself, but to the expected lower punishment in other conditions. Greed led to expected lower punishment, especially if it was to be taken from oneself.

# References

1. Gray, Jeffrey. A. & McNaughton, N. *The Neuropsychology of Anxiety: An Enquiry into the Functions of the Septo-Hippocampal System.* (Oxford University Press, Oxford, 2000).

2. Gray, Jeffrey. A. & McNaughton, N. The neuropsychology of anxiety: reprise. *Neb. Symp. Motiv. Neb. Symp. Motiv.* **43**, 61–134 (1996).

3. Raffety, B. D., Smith, R. E. & Ptacek, J. T. Facilitating and debilitating trait anxiety, situational anxiety, and coping with an anticipated stressor: A process analysis. *J. Pers. Soc. Psychol.* **72**, 892–906 (1997).

4. Turner, J. H. *A Theory of Social Interaction*. (Stanford University Press, Stanford, CA, 1988).

5. Wu, T., Luo, Y., Broster, L. S., Gu, R. & Luo, Y. The impact of anxiety on social decision-making: Behavioral and electrodermal findings. *Soc. Neurosci.* **8**, 11–21 (2013).

6. Luo, Y. *et al.* The temporal course of the influence of anxiety on fairness considerations. *Psychophysiology* **51**, 834–842 (2014).

7. Wray, L. D. & Stone, E. R. The role of self-esteem and anxiety in decision making for self versus others in relationships. *J. Behav. Decis. Mak.* **18**, 125–144 (2005).

8. Seuntjens, T. G., Zeelenberg, M., Van De Ven, N. & Breugelmans, S. M. Dispositional greed. *J. Pers. Soc. Psychol.* **108**, 917–933 (2015).

9. Mussel, P., Rodrigues, J., Krumm, S. & Hewig, J. The convergent validity of five dispositional greed scales. *Personal. Individ. Differ.* **131**, 249–253 (2018).

10. Mussel, P., Reiter, A. M. F., Osinsky, R. & Hewig, J. State- and trait-greed, its impact on risky decision-making and underlying neural mechanisms. *Soc. Neurosci.* **10**, 126–134 (2015).

11. Seuntjens, T. G., Zeelenberg, M., van de Ven, N. & Breugelmans, S. M. Greedy bastards: Testing the relationship between wanting more and unethical behavior. *Personal. Individ. Differ.* **138**, 147–156 (2019).

12. Mussel, P. & Hewig, J. The life and times of individuals scoring high and low on dispositional greed. *J. Res. Personal.* **64**, 52–60 (2016).

13. Mussel, P. & Hewig, J. A neural perspective on when and why trait greed comes at the expense of others. *Sci. Rep.* **9**, (2019).

14. Rodrigues, J. *et al.* *You Get What You Deserve! Reactance, Greed and Altruism in the Dictator Game with Offer Suggestions by the Receiver*. (2021) doi:10.31234/OSF.IO/QVZMP.

15. Thielmann, I., Spadaro, G. & Balliet, D. Personality and prosocial behavior: A theoretical framework and meta-analysis. *Psychol. Bull.* **146**, 30–90 (2020).

16. Seuntjens, T. G., Zeelenberg, M., van de Ven, N. & Breugelmans, S. M. Greedy bastards: Testing the relationship between wanting more and unethical behavior. *Personal. Individ. Differ.* **138**, 147–156 (2019).

17. Watson, D., Clark, L. A. & Tellegen, A. Development and validation of brief measures of positive and negative affect: the PANAS scales. *J. Pers. Soc. Psychol.* **54**, 1063–1070 (1988).

18. Krohne, Heinz, W., Egloff, B., Kohlmann, C.-W. & Tausch, A. Untersuchungen mit einer deutschen Version der ‘Positive and Negative Affect Schedule’ (PANAS). *Diagnostica* **42**, 139–156 (1996).

19. Bradley, M. M. & Lang, P. J. Measuring emotion: The self-assessment manikin and the semantic differential. *J. Behav. Ther. Exp. Psychiatry* **25**, 49–59 (1994).

20. Laux, L., Glanzmann, P., Schaffner, P. & Spielberger, C. D. Das state-trait-angstinventar [The state-trait anxiety inventory]. *Hogrefe Gött. Ger.* (1981).

21. DeYoung, C. G., Quilty, L. C. & Peterson, J. B. Between facets and domains: 10 aspects of the Big Five. *J. Pers. Soc. Psychol.* **93**, 880–896 (2007).

22. Takagishi, H., Fujii, T., Nishina, K. & Okada, H. Fear of negative evaluation moderates the effect of subliminal fear priming on rejection of unfair offers in the ultimatum game. *Sci. Rep.* **6**, (2016).

23. Maner, J. K. *et al.* Dispositional anxiety and risk-avoidant decision-making. *Personal. Individ. Differ.* **42**, 665–675 (2007).

24. Rodrigues, J. *et al.* You get what you deserve! Reactance, greed and altruism in the dictator game with offer suggestions by the receiver. *Personal. Individ. Differ.* **185**, 111271 (2022).
