# Supplementary material for: The relationship of the source of punishment and personality traits with investment and punishment in a public goods game
Source: Sci Rep. 2024 Sep 9;14:21046. doi: 10.1038/s41598-024-71106-x (PMC11385193; doi:10.1038/s41598-024-71106-x)
Supplement: Supplementary file 6 — Supplementary Table S6. [file 41598_2024_71106_MOESM6_ESM.docx]

# Supplemental materials S6: Results in details:

| **Table S6a: Correlations of the traits altruism, anger, empathy, anxiety and greed** | | | | | |
| --- | --- | --- | --- | --- | --- |
|  |  | **altruism** | **anger** | **anxiety** | **empathy** |
| **anger** | Pearson's r | -0.219 | — |  |  |
|  | p-value | 0.023 | — |  |  |
| **anxiety** | Pearson's r | -0.083 | 0.323 | — |  |
|  | p-value | 0.397 | < .001 | — |  |
| **empathy** | Pearson's r | 0.36 | -0.018 | 0.068 | — |
|  | p-value | < .001 | 0.857 | 0.485 | — |
| **greed** | Pearson's r | -0.39 | 0.52 | 0.114 | -0.159 |
|  | p-value | < .001 | < .001 | 0.244 | 0.101 |

| **Table S6b: Model parameter estimation: Amount of punishment** |  |  |  |  |  |
| --- | --- | --- | --- | --- | --- |
|  | **Estimate** | **Std.Error** | **z-value** | **Pr(>\|z\|)** |  |
| (Intercept) | 4.585 | 0.368 | 12.455 | 0.000 | *** |
| Blockpunish_pool | 0.820 | 0.112 | 7.299 | 0.000 | *** |
| Blockpunish_self | -3.243 | 0.116 | -27.986 | 0.000 | *** |
| Cinvestother_total | -0.097 | 0.007 | -13.997 | 0.000 | *** |
| investself.gc | 0.438 | 0.021 | 20.466 | 0.000 | *** |
| CTrials | -0.008 | 0.004 | -1.783 | 0.075 | . |
| meanInvestself.gmc | -0.007 | 0.125 | -0.057 | 0.954 |  |
| CAltruism_Carlo | -1.284 | 0.841 | -1.526 | 0.127 |  |
| CGreed | -0.309 | 0.431 | -0.716 | 0.474 |  |
| CSTAXI_Total | 1.408 | 1.116 | 1.261 | 0.207 |  |
| CSTAI_Total | -0.911 | 0.882 | -1.034 | 0.301 |  |
| Cempathy | 0.013 | 0.055 | 0.243 | 0.808 |  |
| Blockpunish_pool:Cinvestother_total | -0.019 | 0.010 | -1.915 | 0.055 | . |
| Blockpunish_self:Cinvestother_total | 0.055 | 0.010 | 5.445 | 0.000 | *** |
| Blockpunish_pool:investself.gc | 0.134 | 0.032 | 4.223 | 0.000 | *** |
| Blockpunish_self:investself.gc | -0.323 | 0.033 | -9.866 | 0.000 | *** |
| Cinvestother_total:investself.gc | -0.026 | 0.002 | -11.174 | 0.000 | *** |
| Blockpunish_pool:CTrials | 0.006 | 0.003 | 2.003 | 0.045 | * |
| Blockpunish_self:CTrials | -0.008 | 0.003 | -2.500 | 0.012 | * |
| Cinvestother_total:CTrials | 0.000 | 0.000 | -0.546 | 0.585 |  |
| investself.gc:CTrials | 0.002 | 0.000 | 4.968 | 0.000 | *** |
| Blockpunish_pool:meanInvestself.gmc | -0.317 | 0.040 | -7.979 | 0.000 | *** |
| Blockpunish_self:meanInvestself.gmc | 0.047 | 0.039 | 1.210 | 0.226 |  |
| Cinvestother_total:meanInvestself.gmc | -0.045 | 0.002 | -18.914 | 0.000 | *** |
| investself.gc:meanInvestself.gmc | 0.039 | 0.008 | 4.791 | 0.000 | *** |
| CTrials:meanInvestself.gmc | 0.001 | 0.002 | 0.623 | 0.533 |  |
| Blockpunish_pool:CAltruism_Carlo | 1.344 | 0.254 | 5.304 | 0.000 | *** |
| Blockpunish_self:CAltruism_Carlo | 0.278 | 0.285 | 0.977 | 0.329 |  |
| Cinvestother_total:CAltruism_Carlo | -0.080 | 0.016 | -5.042 | 0.000 | *** |
| investself.gc:CAltruism_Carlo | -0.166 | 0.042 | -3.948 | 0.000 | *** |
| CTrials:CAltruism_Carlo | 0.017 | 0.010 | 1.716 | 0.086 | . |
| Blockpunish_pool:CGreed | -0.133 | 0.154 | -0.868 | 0.385 |  |
| Blockpunish_self:CGreed | -0.306 | 0.130 | -2.355 | 0.019 | * |
| Cinvestother_total:CGreed | -0.019 | 0.008 | -2.379 | 0.017 | * |
| investself.gc:CGreed | -0.028 | 0.024 | -1.176 | 0.240 |  |
| CTrials:CGreed | -0.004 | 0.005 | -0.741 | 0.459 |  |
| Blockpunish_pool:CSTAXI_Total | -0.579 | 0.362 | -1.598 | 0.110 |  |
| Blockpunish_self:CSTAXI_Total | -1.665 | 0.343 | -4.856 | 0.000 | *** |
| Cinvestother_total:CSTAXI_Total | -0.037 | 0.021 | -1.735 | 0.083 | . |
| investself.gc:CSTAXI_Total | -0.181 | 0.057 | -3.177 | 0.001 | ** |
| CTrials:CSTAXI_Total | -0.017 | 0.014 | -1.230 | 0.219 |  |
| Blockpunish_pool:CSTAI_Total | 0.363 | 0.270 | 1.343 | 0.179 |  |
| Blockpunish_self:CSTAI_Total | 1.720 | 0.274 | 6.284 | 0.000 | *** |
| Cinvestother_total:CSTAI_Total | -0.005 | 0.017 | -0.287 | 0.774 |  |
| investself.gc:CSTAI_Total | 0.148 | 0.052 | 2.838 | 0.005 | ** |
| CTrials:CSTAI_Total | 0.036 | 0.011 | 3.362 | 0.001 | *** |
| Blockpunish_pool:Cempathy | -0.220 | 0.018 | -12.501 | 0.000 | *** |
| Blockpunish_self:Cempathy | -0.042 | 0.019 | -2.235 | 0.025 | * |
| Cinvestother_total:Cempathy | -0.002 | 0.001 | -2.082 | 0.037 | * |
| investself.gc:Cempathy | -0.006 | 0.003 | -2.073 | 0.038 | * |
| CTrials:Cempathy | -0.003 | 0.001 | -5.177 | 0.000 | *** |
| Blockpunish_pool:Cinvestother_total:investself.gc | -0.008 | 0.004 | -2.184 | 0.029 | * |
| Blockpunish_self:Cinvestother_total:investself.gc | 0.018 | 0.004 | 5.109 | 0.000 | *** |
| Blockpunish_pool:Cinvestother_total:CTrials | 0.000 | 0.000 | 0.437 | 0.662 |  |
| Blockpunish_self:Cinvestother_total:CTrials | 0.000 | 0.000 | 0.246 | 0.806 |  |
| Blockpunish_pool:investself.gc:CTrials | -0.003 | 0.001 | -4.417 | 0.000 | *** |
| Blockpunish_self:investself.gc:CTrials | -0.003 | 0.001 | -3.442 | 0.001 | *** |
| Cinvestother_total:investself.gc:CTrials | 0.000 | 0.000 | 0.887 | 0.375 |  |
| Blockpunish_pool:Cinvestother_total:meanInvestself.gmc | -0.005 | 0.003 | -1.572 | 0.116 |  |
| Blockpunish_self:Cinvestother_total:meanInvestself.gmc | 0.035 | 0.003 | 10.154 | 0.000 | *** |
| Blockpunish_pool:investself.gc:meanInvestself.gmc | -0.063 | 0.012 | -5.335 | 0.000 | *** |
| Blockpunish_self:investself.gc:meanInvestself.gmc | -0.005 | 0.012 | -0.454 | 0.650 |  |
| Cinvestother_total:investself.gc:meanInvestself.gmc | 0.000 | 0.001 | -0.122 | 0.903 |  |
| Blockpunish_pool:CTrials:meanInvestself.gmc | -0.001 | 0.001 | -0.630 | 0.529 |  |
| Blockpunish_self:CTrials:meanInvestself.gmc | 0.001 | 0.001 | 0.708 | 0.479 |  |
| Cinvestother_total:CTrials:meanInvestself.gmc | 0.000 | 0.000 | 0.128 | 0.898 |  |
| investself.gc:CTrials:meanInvestself.gmc | -0.001 | 0.000 | -5.107 | 0.000 | *** |
| Blockpunish_pool:Cinvestother_total:CAltruism_Carlo | -0.004 | 0.022 | -0.195 | 0.845 |  |
| Blockpunish_self:Cinvestother_total:CAltruism_Carlo | 0.099 | 0.023 | 4.229 | 0.000 | *** |
| Blockpunish_pool:investself.gc:CAltruism_Carlo | 0.397 | 0.061 | 6.460 | 0.000 | *** |
| Blockpunish_self:investself.gc:CAltruism_Carlo | 0.318 | 0.066 | 4.782 | 0.000 | *** |
| Cinvestother_total:investself.gc:CAltruism_Carlo | 0.007 | 0.005 | 1.413 | 0.158 |  |
| Blockpunish_pool:CTrials:CAltruism_Carlo | 0.014 | 0.007 | 1.881 | 0.060 | . |
| Blockpunish_self:CTrials:CAltruism_Carlo | 0.009 | 0.007 | 1.248 | 0.212 |  |
| Cinvestother_total:CTrials:CAltruism_Carlo | 0.000 | 0.000 | 0.065 | 0.948 |  |
| investself.gc:CTrials:CAltruism_Carlo | 0.006 | 0.001 | 6.770 | 0.000 | *** |
| Blockpunish_pool:Cinvestother_total:CGreed | 0.012 | 0.011 | 1.013 | 0.311 |  |
| Blockpunish_self:Cinvestother_total:CGreed | 0.038 | 0.012 | 3.187 | 0.001 | ** |
| Blockpunish_pool:investself.gc:CGreed | 0.129 | 0.036 | 3.591 | 0.000 | *** |
| Blockpunish_self:investself.gc:CGreed | 0.032 | 0.036 | 0.869 | 0.385 |  |
| Cinvestother_total:investself.gc:CGreed | 0.002 | 0.003 | 0.885 | 0.376 |  |
| Blockpunish_pool:CTrials:CGreed | 0.021 | 0.003 | 6.270 | 0.000 | *** |
| Blockpunish_self:CTrials:CGreed | 0.013 | 0.004 | 3.176 | 0.001 | ** |
| Cinvestother_total:CTrials:CGreed | 0.000 | 0.000 | -1.306 | 0.192 |  |
| investself.gc:CTrials:CGreed | 0.001 | 0.000 | 1.726 | 0.084 | . |
| Blockpunish_pool:Cinvestother_total:CSTAXI_Total | 0.065 | 0.030 | 2.181 | 0.029 | * |
| Blockpunish_self:Cinvestother_total:CSTAXI_Total | 0.013 | 0.030 | 0.439 | 0.661 |  |
| Blockpunish_pool:investself.gc:CSTAXI_Total | -0.041 | 0.089 | -0.457 | 0.648 |  |
| Blockpunish_self:investself.gc:CSTAXI_Total | 0.260 | 0.089 | 2.911 | 0.004 | ** |
| Cinvestother_total:investself.gc:CSTAXI_Total | -0.006 | 0.006 | -0.957 | 0.339 |  |
| Blockpunish_pool:CTrials:CSTAXI_Total | 0.011 | 0.009 | 1.186 | 0.236 |  |
| Blockpunish_self:CTrials:CSTAXI_Total | -0.015 | 0.010 | -1.536 | 0.125 |  |
| Cinvestother_total:CTrials:CSTAXI_Total | 0.000 | 0.000 | 0.335 | 0.738 |  |
| investself.gc:CTrials:CSTAXI_Total | -0.001 | 0.001 | -0.815 | 0.415 |  |
| Blockpunish_pool:Cinvestother_total:CSTAI_Total | -0.022 | 0.023 | -0.951 | 0.342 |  |
| Blockpunish_self:Cinvestother_total:CSTAI_Total | 0.009 | 0.024 | 0.378 | 0.705 |  |
| Blockpunish_pool:investself.gc:CSTAI_Total | -0.272 | 0.074 | -3.694 | 0.000 | *** |
| Blockpunish_self:investself.gc:CSTAI_Total | -0.156 | 0.078 | -1.996 | 0.046 | * |
| Cinvestother_total:investself.gc:CSTAI_Total | 0.002 | 0.006 | 0.335 | 0.738 |  |
| Blockpunish_pool:CTrials:CSTAI_Total | -0.046 | 0.007 | -6.286 | 0.000 | *** |
| Blockpunish_self:CTrials:CSTAI_Total | -0.046 | 0.007 | -6.199 | 0.000 | *** |
| Cinvestother_total:CTrials:CSTAI_Total | 0.001 | 0.000 | 2.317 | 0.020 | * |
| investself.gc:CTrials:CSTAI_Total | -0.003 | 0.001 | -2.958 | 0.003 | ** |
| Blockpunish_pool:Cinvestother_total:Cempathy | -0.002 | 0.001 | -1.394 | 0.163 |  |
| Blockpunish_self:Cinvestother_total:Cempathy | 0.002 | 0.002 | 1.590 | 0.112 |  |
| Blockpunish_pool:investself.gc:Cempathy | 0.006 | 0.004 | 1.624 | 0.104 |  |
| Blockpunish_self:investself.gc:Cempathy | 0.003 | 0.004 | 0.667 | 0.505 |  |
| Cinvestother_total:investself.gc:Cempathy | -0.001 | 0.000 | -4.087 | 0.000 | *** |
| Blockpunish_pool:CTrials:Cempathy | 0.004 | 0.000 | 9.592 | 0.000 | *** |
| Blockpunish_self:CTrials:Cempathy | 0.003 | 0.000 | 6.028 | 0.000 | *** |
| Cinvestother_total:CTrials:Cempathy | 0.000 | 0.000 | -0.776 | 0.438 |  |
| investself.gc:CTrials:Cempathy | 0.000 | 0.000 | -0.712 | 0.476 |  |
| Blockpunish_pool:Cinvestother_total:investself.gc:CTrials | 0.000 | 0.000 | -0.851 | 0.395 |  |
| Blockpunish_self:Cinvestother_total:investself.gc:CTrials | 0.000 | 0.000 | -1.758 | 0.079 | . |
| Blockpunish_pool:Cinvestother_total:investself.gc:meanInvestself.gmc | 0.004 | 0.001 | 2.726 | 0.006 | ** |
| Blockpunish_self:Cinvestother_total:investself.gc:meanInvestself.gmc | 0.001 | 0.001 | 0.585 | 0.558 |  |
| Blockpunish_pool:Cinvestother_total:CTrials:meanInvestself.gmc | 0.000 | 0.000 | 0.570 | 0.569 |  |
| Blockpunish_self:Cinvestother_total:CTrials:meanInvestself.gmc | 0.000 | 0.000 | 1.016 | 0.310 |  |
| Blockpunish_pool:investself.gc:CTrials:meanInvestself.gmc | 0.000 | 0.000 | -1.044 | 0.297 |  |
| Blockpunish_self:investself.gc:CTrials:meanInvestself.gmc | 0.001 | 0.000 | 4.501 | 0.000 | *** |
| Cinvestother_total:investself.gc:CTrials:meanInvestself.gmc | 0.000 | 0.000 | -0.788 | 0.431 |  |
| Blockpunish_pool:Cinvestother_total:investself.gc:CAltruism_Carlo | -0.015 | 0.007 | -2.081 | 0.037 | * |
| Blockpunish_self:Cinvestother_total:investself.gc:CAltruism_Carlo | -0.005 | 0.007 | -0.702 | 0.482 |  |
| Blockpunish_pool:Cinvestother_total:CTrials:CAltruism_Carlo | 0.002 | 0.000 | 4.215 | 0.000 | *** |
| Blockpunish_self:Cinvestother_total:CTrials:CAltruism_Carlo | 0.001 | 0.000 | 1.892 | 0.059 | . |
| Blockpunish_pool:investself.gc:CTrials:CAltruism_Carlo | -0.013 | 0.001 | -9.270 | 0.000 | *** |
| Blockpunish_self:investself.gc:CTrials:CAltruism_Carlo | -0.004 | 0.001 | -3.070 | 0.002 | ** |
| Cinvestother_total:investself.gc:CTrials:CAltruism_Carlo | 0.000 | 0.000 | -4.029 | 0.000 | *** |
| Blockpunish_pool:Cinvestother_total:investself.gc:CGreed | -0.005 | 0.004 | -1.357 | 0.175 |  |
| Blockpunish_self:Cinvestother_total:investself.gc:CGreed | 0.000 | 0.004 | -0.102 | 0.919 |  |
| Blockpunish_pool:Cinvestother_total:CTrials:CGreed | 0.000 | 0.000 | 2.150 | 0.032 | * |
| Blockpunish_self:Cinvestother_total:CTrials:CGreed | 0.000 | 0.000 | 1.691 | 0.091 | . |
| Blockpunish_pool:investself.gc:CTrials:CGreed | 0.000 | 0.001 | -0.245 | 0.807 |  |
| Blockpunish_self:investself.gc:CTrials:CGreed | 0.000 | 0.001 | 0.177 | 0.860 |  |
| Cinvestother_total:investself.gc:CTrials:CGreed | 0.000 | 0.000 | -0.536 | 0.592 |  |
| Blockpunish_pool:Cinvestother_total:investself.gc:CSTAXI_Total | 0.001 | 0.010 | 0.105 | 0.917 |  |
| Blockpunish_self:Cinvestother_total:investself.gc:CSTAXI_Total | 0.010 | 0.010 | 0.956 | 0.339 |  |
| Blockpunish_pool:Cinvestother_total:CTrials:CSTAXI_Total | 0.000 | 0.001 | 0.309 | 0.757 |  |
| Blockpunish_self:Cinvestother_total:CTrials:CSTAXI_Total | -0.001 | 0.001 | -0.982 | 0.326 |  |
| Blockpunish_pool:investself.gc:CTrials:CSTAXI_Total | 0.003 | 0.002 | 1.431 | 0.152 |  |
| Blockpunish_self:investself.gc:CTrials:CSTAXI_Total | 0.001 | 0.002 | 0.647 | 0.518 |  |
| Cinvestother_total:investself.gc:CTrials:CSTAXI_Total | 0.000 | 0.000 | 2.783 | 0.005 | ** |
| Blockpunish_pool:Cinvestother_total:investself.gc:CSTAI_Total | -0.025 | 0.008 | -3.044 | 0.002 | ** |
| Blockpunish_self:Cinvestother_total:investself.gc:CSTAI_Total | -0.007 | 0.009 | -0.796 | 0.426 |  |
| Blockpunish_pool:Cinvestother_total:CTrials:CSTAI_Total | -0.002 | 0.000 | -3.416 | 0.001 | *** |
| Blockpunish_self:Cinvestother_total:CTrials:CSTAI_Total | -0.001 | 0.001 | -1.873 | 0.061 | . |
| Blockpunish_pool:investself.gc:CTrials:CSTAI_Total | 0.002 | 0.002 | 1.581 | 0.114 |  |
| Blockpunish_self:investself.gc:CTrials:CSTAI_Total | 0.002 | 0.002 | 1.173 | 0.241 |  |
| Cinvestother_total:investself.gc:CTrials:CSTAI_Total | 0.000 | 0.000 | -2.364 | 0.018 | * |
| Blockpunish_pool:Cinvestother_total:investself.gc:Cempathy | 0.000 | 0.000 | 1.081 | 0.280 |  |
| Blockpunish_self:Cinvestother_total:investself.gc:Cempathy | 0.002 | 0.000 | 3.464 | 0.001 | *** |
| Blockpunish_pool:Cinvestother_total:CTrials:Cempathy | 0.000 | 0.000 | -0.174 | 0.862 |  |
| Blockpunish_self:Cinvestother_total:CTrials:Cempathy | 0.000 | 0.000 | -1.145 | 0.252 |  |
| Blockpunish_pool:investself.gc:CTrials:Cempathy | 0.000 | 0.000 | 2.358 | 0.018 | * |
| Blockpunish_self:investself.gc:CTrials:Cempathy | 0.000 | 0.000 | 2.806 | 0.005 | ** |
| Cinvestother_total:investself.gc:CTrials:Cempathy | 0.000 | 0.000 | 4.979 | 0.000 | *** |
| Blockpunish_pool:Cinvestother_total:investself.gc:CTrials:meanInvestself.gmc | 0.000 | 0.000 | 0.694 | 0.488 |  |
| Blockpunish_self:Cinvestother_total:investself.gc:CTrials:meanInvestself.gmc | 0.000 | 0.000 | 1.650 | 0.099 | . |
| Blockpunish_pool:Cinvestother_total:investself.gc:CTrials:CAltruism_Carlo | 0.001 | 0.000 | 4.214 | 0.000 | *** |
| Blockpunish_self:Cinvestother_total:investself.gc:CTrials:CAltruism_Carlo | 0.000 | 0.000 | 2.698 | 0.007 | ** |
| Blockpunish_pool:Cinvestother_total:investself.gc:CTrials:CGreed | 0.000 | 0.000 | -0.642 | 0.521 |  |
| Blockpunish_self:Cinvestother_total:investself.gc:CTrials:CGreed | 0.000 | 0.000 | 1.462 | 0.144 |  |
| Blockpunish_pool:Cinvestother_total:investself.gc:CTrials:CSTAXI_Total | -0.001 | 0.000 | -3.218 | 0.001 | ** |
| Blockpunish_self:Cinvestother_total:investself.gc:CTrials:CSTAXI_Total | -0.001 | 0.000 | -2.552 | 0.011 | * |
| Blockpunish_pool:Cinvestother_total:investself.gc:CTrials:CSTAI_Total | 0.000 | 0.000 | 1.854 | 0.064 | . |
| Blockpunish_self:Cinvestother_total:investself.gc:CTrials:CSTAI_Total | 0.000 | 0.000 | -0.055 | 0.956 |  |
| Blockpunish_pool:Cinvestother_total:investself.gc:CTrials:Cempathy | 0.000 | 0.000 | -2.433 | 0.015 | * |
| Blockpunish_self:Cinvestother_total:investself.gc:CTrials:Cempathy | 0.000 | 0.000 | -3.442 | 0.001 | *** |

| **Table S6c: Model parameter estimation: Source of punishment** | | | |  | |  | |  | |
| --- | --- | --- | --- | --- | --- | --- | --- | --- | --- |
|  | **Estimate** | **Std.Error** | **z-value** | | **Pr(>\|z\|)** | |  | |  |
| (Intercept) | 1.859 | 0.266 | 6.997 | | 0.000 | | *** | |  |
| Cinvestother_total | -0.042 | 0.005 | -8.501 | | 0.000 | | *** | |  |
| InvestSelf.gc | 0.108 | 0.016 | 6.681 | | 0.000 | | *** | |  |
| meanInvestSelf.gmc | -0.013 | 0.078 | -0.161 | | 0.872 | |  | |  |
| CAltruism_Carlo | 0.422 | 0.512 | 0.825 | | 0.409 | |  | |  |
| Cinvestother_total:InvestSelf.gc | -0.002 | 0.002 | -0.901 | | 0.367 | |  | |  |
| Cinvestother_total:meanInvestSelf.gmc | -0.004 | 0.002 | -2.184 | | 0.029 | | * | |  |
| InvestSelf.gc:meanInvestSelf.gmc | -0.002 | 0.006 | -0.303 | | 0.762 | |  | |  |
| Cinvestother_total:CAltruism_Carlo | -0.033 | 0.010 | -3.231 | | 0.001 | | ** | |  |
| InvestSelf.gc:CAltruism_Carlo | -0.020 | 0.028 | -0.716 | | 0.474 | |  | |  |
| Cinvestother_total:InvestSelf.gc:meanInvestSelf.gmc | 0.001 | 0.001 | 2.124 | | 0.034 | | * | |  |
| Cinvestother_total:InvestSelf.gc:CAltruism_Carlo | 0.004 | 0.003 | 1.254 | | 0.210 | |  | |  |

| **Table S6d: Model parameter estimation: Mouse trajectory** | |  | | |  | |  | | |  |  |
| --- | --- | --- | --- | --- | --- | --- | --- | --- | --- | --- | --- |
|  | **Estimate** | | **Std.Error** | **z-value** | | **Pr(>\|z\|)** | |  |  |  |  |
| (Intercept) | -0.004 | | 0.002 | -1.748 | | 0.081 | | . |  |  |  |
| Cinvestother_total | 0.000 | | 0.000 | 1.129 | | 0.259 | |  |  |  |  |
| PunFac1 | 0.005 | | 0.002 | 2.140 | | 0.032 | | * |  |  |  |
| InvestSelf.gc | -0.002 | | 0.001 | -2.362 | | 0.018 | | * |  |  |  |
| meanInvestSelf.gmc | -0.001 | | 0.001 | -0.919 | | 0.358 | |  |  |  |  |
| CAltruism_Carlo | -0.003 | | 0.004 | -0.664 | | 0.507 | |  |  |  |  |
| CGreed | 0.001 | | 0.003 | 0.530 | | 0.596 | |  |  |  |  |
| CSTAXI_Total | 0.008 | | 0.007 | 1.122 | | 0.262 | |  |  |  |  |
| CSTAI_Total | 0.005 | | 0.005 | 0.992 | | 0.321 | |  |  |  |  |
| Cempathy | 0.000 | | 0.000 | -1.316 | | 0.188 | |  |  |  |  |
| Cinvestother_total:PunFac1 | -0.001 | | 0.000 | -1.989 | | 0.047 | | * |  |  |  |
| Cinvestother_total:InvestSelf.gc | 0.000 | | 0.000 | 1.267 | | 0.205 | |  |  |  |  |
| PunFac1:InvestSelf.gc | 0.002 | | 0.001 | 2.624 | | 0.009 | | ** |  |  |  |
| Cinvestother_total:meanInvestSelf.gmc | 0.000 | | 0.000 | -0.497 | | 0.619 | |  |  |  |  |
| PunFac1:meanInvestSelf.gmc | 0.000 | | 0.001 | 0.576 | | 0.564 | |  |  |  |  |
| InvestSelf.gc:meanInvestSelf.gmc | 0.000 | | 0.000 | -0.314 | | 0.754 | |  |  |  |  |
| Cinvestother_total:CAltruism_Carlo | 0.000 | | 0.000 | 1.138 | | 0.255 | |  |  |  |  |
| PunFac1:CAltruism_Carlo | -0.001 | | 0.005 | -0.289 | | 0.773 | |  |  |  |  |
| InvestSelf.gc:CAltruism_Carlo | 0.000 | | 0.001 | -0.201 | | 0.841 | |  |  |  |  |
| Cinvestother_total:CGreed | 0.000 | | 0.000 | -0.584 | | 0.559 | |  |  |  |  |
| PunFac1:CGreed | 0.001 | | 0.003 | 0.366 | | 0.714 | |  |  |  |  |
| InvestSelf.gc:CGreed | 0.001 | | 0.001 | 1.229 | | 0.219 | |  |  |  |  |
| Cinvestother_total:CSTAXI_Total | 0.001 | | 0.001 | 1.267 | | 0.205 | |  |  |  |  |
| PunFac1:CSTAXI_Total | -0.007 | | 0.008 | -0.921 | | 0.357 | |  |  |  |  |
| InvestSelf.gc:CSTAXI_Total | -0.001 | | 0.002 | -0.464 | | 0.642 | |  |  |  |  |
| Cinvestother_total:CSTAI_Total | 0.000 | | 0.001 | 0.513 | | 0.608 | |  |  |  |  |
| PunFac1:CSTAI_Total | -0.013 | | 0.006 | -2.288 | | 0.022 | | * |  |  |  |
| InvestSelf.gc:CSTAI_Total | -0.001 | | 0.001 | -0.746 | | 0.455 | |  |  |  |  |
| Cinvestother_total:Cempathy | 0.000 | | 0.000 | -0.602 | | 0.547 | |  |  |  |  |
| PunFac1:Cempathy | 0.001 | | 0.000 | 3.204 | | 0.001 | | ** |  |  |  |
| InvestSelf.gc:Cempathy | 0.000 | | 0.000 | -1.018 | | 0.309 | |  |  |  |  |
| Cinvestother_total:PunFac1:InvestSelf.gc | -0.0002 | | 0.000 | -2.216 | | 0.027 | | * |  |  |  |
| Cinvestother_total:PunFac1:meanInvestSelf.gmc | 0.000 | | 0.000 | -0.552 | | 0.581 | |  |  |  |  |
| Cinvestother_total:InvestSelf.gc:meanInvestSelf.gmc | 0.000 | | 0.000 | 0.692 | | 0.489 | |  |  |  |  |
| PunFac1:InvestSelf.gc:meanInvestSelf.gmc | 0.000 | | 0.000 | 0.977 | | 0.329 | |  |  |  |  |
| Cinvestother_total:PunFac1:CAltruism_Carlo | -0.001 | | 0.001 | -1.495 | | 0.135 | |  |  |  |  |
| Cinvestother_total:InvestSelf.gc:CAltruism_Carlo | 0.000 | | 0.000 | 0.434 | | 0.664 | |  |  |  |  |
| PunFac1:InvestSelf.gc:CAltruism_Carlo | 0.001 | | 0.001 | 0.692 | | 0.489 | |  |  |  |  |
| Cinvestother_total:PunFac1:CGreed | 0.000 | | 0.000 | -0.419 | | 0.675 | |  |  |  |  |
| Cinvestother_total:InvestSelf.gc:CGreed | 0.000 | | 0.000 | 0.640 | | 0.522 | |  |  |  |  |
| PunFac1:InvestSelf.gc:CGreed | 0.000 | | 0.001 | -0.233 | | 0.816 | |  |  |  |  |
| Cinvestother_total:PunFac1:CSTAXI_Total | -0.002 | | 0.001 | -2.733 | | 0.006 | | ** |  |  |  |
| Cinvestother_total:InvestSelf.gc:CSTAXI_Total | 0.000 | | 0.000 | -0.433 | | 0.665 | |  |  |  |  |
| PunFac1:InvestSelf.gc:CSTAXI_Total | 0.003 | | 0.002 | 1.462 | | 0.144 | |  |  |  |  |
| Cinvestother_total:PunFac1:CSTAI_Total | 0.001 | | 0.001 | 1.141 | | 0.254 | |  |  |  |  |
| Cinvestother_total:InvestSelf.gc:CSTAI_Total | 0.000 | | 0.000 | -0.509 | | 0.611 | |  |  |  |  |
| PunFac1:InvestSelf.gc:CSTAI_Total | -0.002 | | 0.002 | -0.948 | | 0.343 | |  |  |  |  |
| Cinvestother_total:PunFac1:Cempathy | 0.000 | | 0.000 | -0.501 | | 0.617 | |  |  |  |  |
| Cinvestother_total:InvestSelf.gc:Cempathy | 0.000 | | 0.000 | 0.301 | | 0.763 | |  |  |  |  |
| PunFac1:InvestSelf.gc:Cempathy | 0.000 | | 0.000 | 1.224 | | 0.221 | |  |  |  |  |
| Cinvestother_total:PunFac1:InvestSelf.gc:meanInvestSelf.gmc | 0.000 | | 0.000 | -1.321 | | 0.187 | |  |  |  |  |
| Cinvestother_total:PunFac1:InvestSelf.gc:CAltruism_Carlo | 0.000 | | 0.000 | -1.272 | | 0.203 | |  |  |  |  |
| Cinvestother_total:PunFac1:InvestSelf.gc:CGreed | 0.000 | | 0.000 | -1.609 | | 0.108 | |  |  |  |  |
| Cinvestother_total:PunFac1:InvestSelf.gc:CSTAXI_Total | -0.0005 | | 0.000 | -1.773 | | 0.076 | | . |  |  |  |
| Cinvestother_total:PunFac1:InvestSelf.gc:CSTAI_Total | 0.0004 | | 0.000 | 2.120 | | 0.034 | | * |  |  |  |
| Cinvestother_total:PunFac1:InvestSelf.gc:Cempathy | 0.000 | | 0.000 | -1.433 | | 0.152 | |  |  |  |  |

| **Table S6e: Model parameter estimation: Investment in the pool** | | |  |  |  |
| --- | --- | --- | --- | --- | --- |
|  | **Estimate** | **Std.Error** | **z-value** | **Pr(>\|z\|)** |  |
| (Intercept) | 5.785 | 0.363 | 15.938 | 0.000 | *** |
| Blockpunish_choice | 1.566 | 0.256 | 6.130 | 0.000 | *** |
| Blockpunish_pool | 1.778 | 0.256 | 6.960 | 0.000 | *** |
| Blockpunish_self | 0.092 | 0.255 | 0.359 | 0.720 |  |
| trials.gc | -0.011 | 0.003 | -3.405 | 0.001 | *** |
| CAltruism_Carlo | -0.456 | 0.822 | -0.555 | 0.579 |  |
| CSTAXI_Total | -3.586 | 1.098 | -3.267 | 0.001 | ** |
| CSTAI_Total | 1.612 | 0.863 | 1.869 | 0.062 | . |
| Cempathy | -0.147 | 0.053 | -2.754 | 0.006 | ** |
| CGreed | -0.501 | 0.423 | -1.183 | 0.237 |  |
| Blockpunish_choice:trials.gc | 0.009 | 0.003 | 2.822 | 0.005 | ** |
| Blockpunish_pool:trials.gc | 0.003 | 0.003 | 0.938 | 0.348 |  |
| Blockpunish_self:trials.gc | 0.003 | 0.003 | 0.988 | 0.323 |  |
| Blockpunish_choice:CAltruism_Carlo | 1.035 | 0.578 | 1.790 | 0.073 | . |
| Blockpunish_pool:CAltruism_Carlo | 1.659 | 0.581 | 2.855 | 0.004 | ** |
| Blockpunish_self:CAltruism_Carlo | 0.951 | 0.577 | 1.648 | 0.099 | . |
| trials.gc:CAltruism_Carlo | -0.004 | 0.007 | -0.572 | 0.567 |  |
| Blockpunish_choice:CSTAXI_Total | 2.793 | 0.776 | 3.597 | 0.000 | *** |
| Blockpunish_pool:CSTAXI_Total | 2.552 | 0.770 | 3.315 | 0.001 | *** |
| Blockpunish_self:CSTAXI_Total | 2.680 | 0.773 | 3.466 | 0.001 | *** |
| trials.gc:CSTAXI_Total | -0.032 | 0.010 | -3.301 | 0.001 | *** |
| Blockpunish_choice:CSTAI_Total | -0.343 | 0.605 | -0.566 | 0.571 |  |
| Blockpunish_pool:CSTAI_Total | -0.682 | 0.605 | -1.127 | 0.260 |  |
| Blockpunish_self:CSTAI_Total | -1.115 | 0.612 | -1.822 | 0.068 | . |
| trials.gc:CSTAI_Total | 0.011 | 0.008 | 1.404 | 0.160 |  |
| Blockpunish_choice:Cempathy | 0.103 | 0.038 | 2.737 | 0.006 | ** |
| Blockpunish_pool:Cempathy | 0.076 | 0.038 | 2.015 | 0.044 | * |
| Blockpunish_self:Cempathy | 0.097 | 0.038 | 2.586 | 0.010 | ** |
| trials.gc:Cempathy | -0.002 | 0.000 | -3.906 | 0.000 | *** |
| Blockpunish_choice:CGreed | 0.367 | 0.298 | 1.232 | 0.218 |  |
| Blockpunish_pool:CGreed | 0.443 | 0.298 | 1.488 | 0.137 |  |
| Blockpunish_self:CGreed | 0.287 | 0.299 | 0.959 | 0.337 |  |
| trials.gc:CGreed | -0.001 | 0.004 | -0.307 | 0.759 |  |
| Blockpunish_choice:trials.gc:CAltruism_Carlo | 0.003 | 0.007 | 0.395 | 0.693 |  |
| Blockpunish_pool:trials.gc:CAltruism_Carlo | 0.005 | 0.007 | 0.613 | 0.540 |  |
| Blockpunish_self:trials.gc:CAltruism_Carlo | 0.012 | 0.007 | 1.642 | 0.101 |  |
| Blockpunish_choice:trials.gc:CSTAXI_Total | 0.045 | 0.010 | 4.600 | 0.000 | *** |
| Blockpunish_pool:trials.gc:CSTAXI_Total | 0.034 | 0.010 | 3.506 | 0.000 | *** |
| Blockpunish_self:trials.gc:CSTAXI_Total | 0.047 | 0.010 | 4.781 | 0.000 | *** |
| Blockpunish_choice:trials.gc:CSTAI_Total | -0.017 | 0.008 | -2.229 | 0.026 | * |
| Blockpunish_pool:trials.gc:CSTAI_Total | -0.010 | 0.008 | -1.329 | 0.184 |  |
| Blockpunish_self:trials.gc:CSTAI_Total | -0.013 | 0.008 | -1.738 | 0.082 | . |
| Blockpunish_choice:trials.gc:Cempathy | 0.001 | 0.000 | 2.328 | 0.020 | * |
| Blockpunish_pool:trials.gc:Cempathy | 0.003 | 0.000 | 5.987 | 0.000 | *** |
| Blockpunish_self:trials.gc:Cempathy | 0.002 | 0.000 | 4.044 | 0.000 | *** |
| Blockpunish_choice:trials.gc:CGreed | 0.005 | 0.004 | 1.272 | 0.204 |  |
| Blockpunish_pool:trials.gc:CGreed | 0.001 | 0.004 | 0.288 | 0.773 |  |
| Blockpunish_self:trials.gc:CGreed | 0.002 | 0.004 | 0.437 | 0.662 |  |
